# In silico analysis of *Anacardium occidentale* phytochemicals: pharmacokinetics, molecular docking, and dynamics of *Cryptococcus neoformans* enzymes

**DOI:** 10.1007/s40203-026-00590-y

**Published:** 2026-02-26

**Authors:** Marcus Vinícius Ferreira da Silva, Jacilene Silva, Matheus Nunes da Rocha, Selene Maia de Morais, Emmanuel Silva Marinho

**Affiliations:** 1 Postgraduate Program in Veterinary Sciences, State University of Ceará, Fortaleza, Ceará Brazil; 2 Department of Biological Chemistry, Regional University of Cariri, Crato, Ceará Brazil; 3 Postgraduate Program in Natural Sciences – PPGCN, State University of Ceará, Fortaleza, Brazil

**Keywords:** Cashew leaves, Antifungal, Mechanism of action, Molecular docking

## Abstract

This study evaluated in silico the antifungal potential of phytochemicals from the leaves of *Anacardium occidentale* (cashew tree) against key enzymatic targets: farnesyltransferase (CnFTase), beta-carbonic anhydrase (β-CA), and adenylosuccinate synthetase (AdSS) from *Cryptococcus neoformans*. Molecular docking simulations were conducted to evaluate the binding affinity of selected compounds to key enzymatic targets. The protein structures were retrieved from the Protein Data Bank (PDB) and prepared using AutoDockTools™, while molecular docking was performed with AutoDockVina. Molecular dynamics simulation was performed using the iMODS server, in order to check the stability as well as mobility in the receptor-ligand complexes following molecular docking. Additionally, ADME-Tox properties were predicted using a consensus approach combining ADMETlab 3.0 and ADMET-AI, assessing parameters such as permeability (PAMPA), metabolism (CYP450), and clearance (*Cl*_int, u_, *Cl*_Micro_, *Cl*_Hepa_). The structural complexity of the ligands was analyzed using the MCE18 score, allowing the identification of compounds with an optimal balance between drug-likeness and synthetic accessibility. Notably, quercetin 3-galactoside, tricetin 3’-xyloside, and kaempferol 4’-glucoside exhibited favorable pharmacokinetic profiles and docking affinities, suggesting their potential as antifungal candidates. A PAMPA profile is estimated for these compounds based on a moderate permeability in more selective cells (High Papp MDCK) and low hepatic clearance, resulting from metabolic stability. Molecular docking studies showed that lead compounds have excellent affinity and specificity for the enzymes CnFTase and AdSS (affinity energy <-6.0 kcal/mol), interacting with the binding sites of the drug Fluconazole. Molecular dynamics simulations indicated a smaller conformational torsion of the Cα of the CnFTase and AdSS structures, suggesting that collective movements for both protein-ligand complexes are stable. The results suggest that these lead compounds are a starting point for new glycosylated drugs inhibiting *Cryptococcus neoformans*.

**Supplementary Information:**

The online version contains supplementary material available at 10.1007/s40203-026-00590-y.

## Introduction

Fungal diseases are infections caused by the overgrowth of pathogenic fungi. Such diseases contaminate many people around the world every year and can cause mild to severe illnesses. They can become invasive, especially for individuals with compromised immune systems, in this case immunosuppressed patients, and can lead to the patient’s death (Quejada et al. 2024; Vitiello et al. 2023). Reports indicate that 90% of invasive fungal infections can be associated with three species of fungi: *Candida* sp., *Cryptococcus* sp., and *Aspergillus* sp. (Deberaldini and Santos 2021). In October 2022, the World Health Organization (WHO) launched the FPPL (Fungal Priority Pathogens List), following the criteria of classification, mortality rate, and distribution. The following fungal pathogens were classified as “critical”: *Cryptococcus neoformans*, *Candida auris*,* Aspergillus fumigatus*, and *Candida albicans*. The classification was based on the following factors: drug resistance, sequelae, annual incidence, and mortality rate (Zhao et al. 2023).

*Cryptococcus neoformans*, classified on the list (FPPL), is an opportunistic pathogen distributed globally and of mainly environmental origin, and is associated with bird droppings, soil, and decaying wood (Zhao et al. 2023). There are several species of *Cryptococcus*; however, two species are distributed worldwide and are related to the number of people infected: *C. neoformans*, found in avian environments, and *C. gattii*, found in some species of trees. However, reports indicate that both species can be found in the same environment, either in bird droppings or in trees (Rathore et al. 2022).

*Cryptococcus* contamination is initiated through the inhalation of infectious spores from environmental sources, leading to initial infection in the lungs and bloodstream. The fungus can disseminate to other organs and the central nervous system (CNS), with the latter being the most critical stage. In this stage, the patient develops cryptococcal meningitis (Tezcan et al. 2023). Current research is focused on the development of a vaccine that can stimulate antibodies to produce defense mechanisms against fungal antigens (Sathiyamoorthy et al. 2024), Conventional treatment involves the administration of fluconazole, amphotericin B, and echinocandins over a medium to long-term period. However, resistance to the fungus and/or toxicity to the patient can occur (De Pereira et al. 2021).

In this perspective, the growing search for alternative medicines is due to the adverse effects of conventional treatments such as antibiotics, antifungals, and antimicrobials. Medicinal plants are essential because they have many therapeutic properties, such as anthelmintic, antimicrobial, anti-inflammatory, antioxidant, immunostimulant, anti-ulcer, and sedative. The use of these herbal medicines can be used in humans as well as in ethnoveterinary, which is the use of medicinal plants for animals (Silva Vidal et al. 2025). Plants can also act as “botanical pesticides” obtained from different parts of the plant to control pathogens in infected environments. Natural compounds such as essential oils, tannins, flavonoids, and phenolic compounds have antimicrobial activity, which makes them effective against pathogens (Shabana et al. 2017).

It is important to note that *Anacardium occidentale*, which belongs to the genus *Anacardium* and the family *Anacardiaceae*, is commonly known as cashew. This plant is found in great abundance in the northeastern region of Brazil and is composed of a fruit (nut) and a pseudo-fruit (cashew). Various parts of the cashew tree have been the subject of scientific study, where extracts have demonstrated antioxidant, anti-inflammatory, anesthetic, bactericidal, and insecticidal properties, as reported by several authors (Souza et al. 2017). Leaves and bark of the cashew tree have demonstrated antimicrobial activity, and the leaves of the cashew tree are employed in folk medicine for gastrointestinal treatment, to treat eczema, psoriasis, scrofula, dyspepsia, genital problems, coughs, intestinal colic, skin diseases related to syphilis and leishmaniasis (Souza et al. 2017).

In order to enhance the process of computer-aided drug discovery, a variety of benchmark classification strategies for selecting lead compounds have been implemented since the development of Lipinski’s Rule of Five (Lipinski 2016). Topological properties, including TPSA, logP, and MW, have been identified as crucial factors influencing the efficacy of pharmaceutical agents during clinical trials assessing cell permeability and oral bioavailability. These properties are intricately linked to lipophilicity and the capacity of small molecules to traverse biological membranes (Lipinski 2004; Veber et al. 2002). Recently, a quantitative drug-likeness estimation criterion, called Medicinal Chemistry Evolution in 2018 (MCE18), has been developed to focus its estimation of drug candidate efficacy on the cumulative attribute of the fraction of sp3 hybridized carbons (Fsp3). It has been frequently posited that structural optimization strategies predicated on the modification of aromatic structures by Fsp3-rich saturated structures are efficacious in enhancing the solubility and permeability of various drugs (Ivanenkov et al. 2019; Wei et al. 2020). Consequently, a multiparametric optimization strategy of pharmacokinetic properties, such as oral bioavailability, as well as bioactivity with more selective targets in the class of enzymes, G protein-coupled receptors (GPCRs), and kinases, has been identified.

In light of the context above, the objective of this study was to undertake an in silico evaluation of the antifungal potential of the phytochemicals present in the cashew tree leaf extract (Sinlapapanya et al. 2022), against *Cryptococcus neoformans*, employing a structure-based virtual screening approach.

## Materials and methods

### Methodological design

**Fig. 1:**
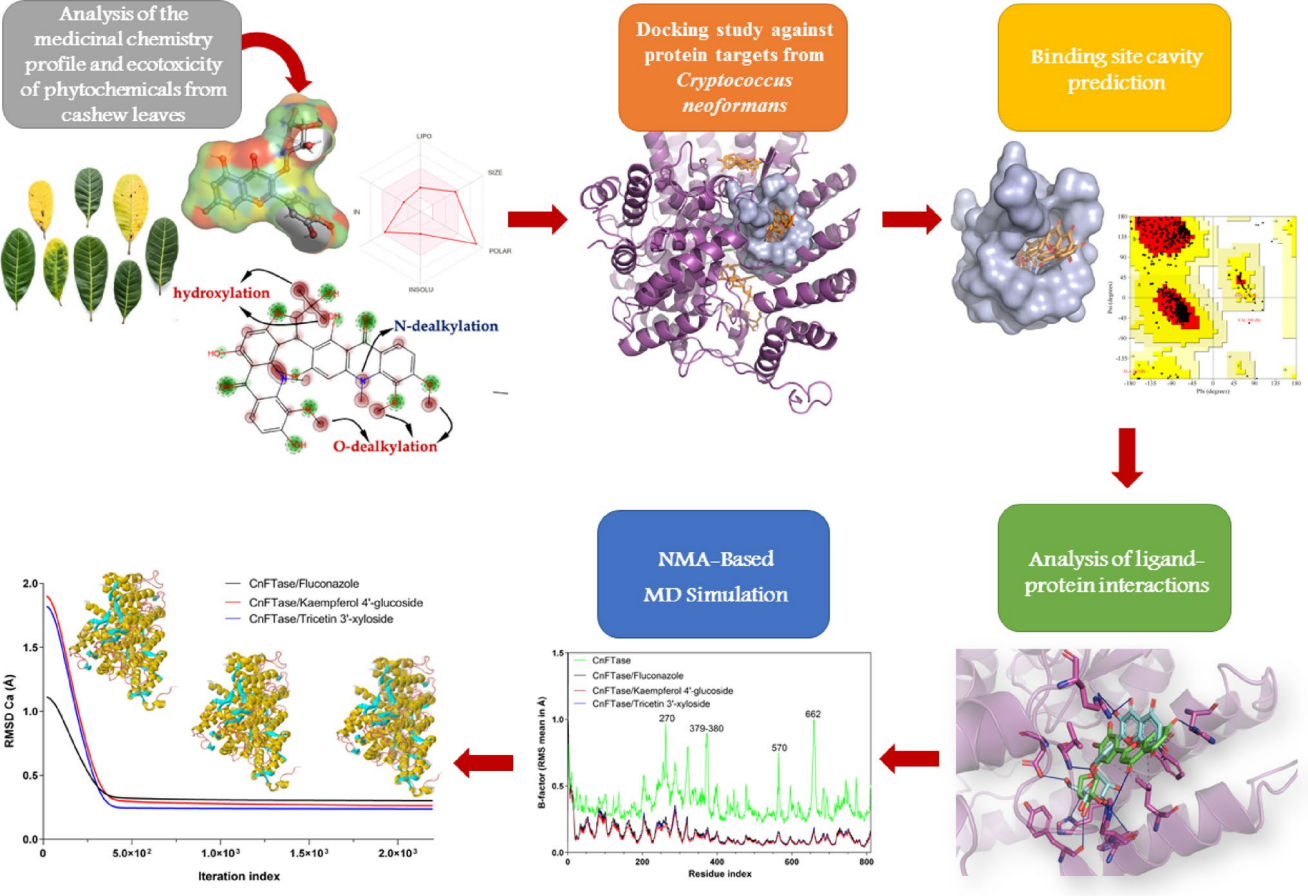
Virtual screening methodological scheme used for this study

The methodological approach entailed the screening of compounds through the use of pharmacokinetic and toxicokinetic predictions, facilitated by specialized software. Subsequently, a ligand-protein interaction analysis was conducted using molecular docking simulations to assess the binding affinity of the compounds to the active site of biomolecular targets relevant to the pathogenicity of the fungus. Additionally, molecular dynamics simulations for analyzing the molecular motion in addition to the structural flexibility via NMA (Normal mode analysis). The results were then subjected to a comprehensive study, encompassing molecular interaction parameters, affinity energy, structural flexibility, and pharmacokinetic properties. This analytical approach was undertaken to identify compounds that exhibited potential for further experimental investigation (Fig. 1) (de Oliveira et al. 2024).

### Obtaining the dataset

**Fig. 2:**
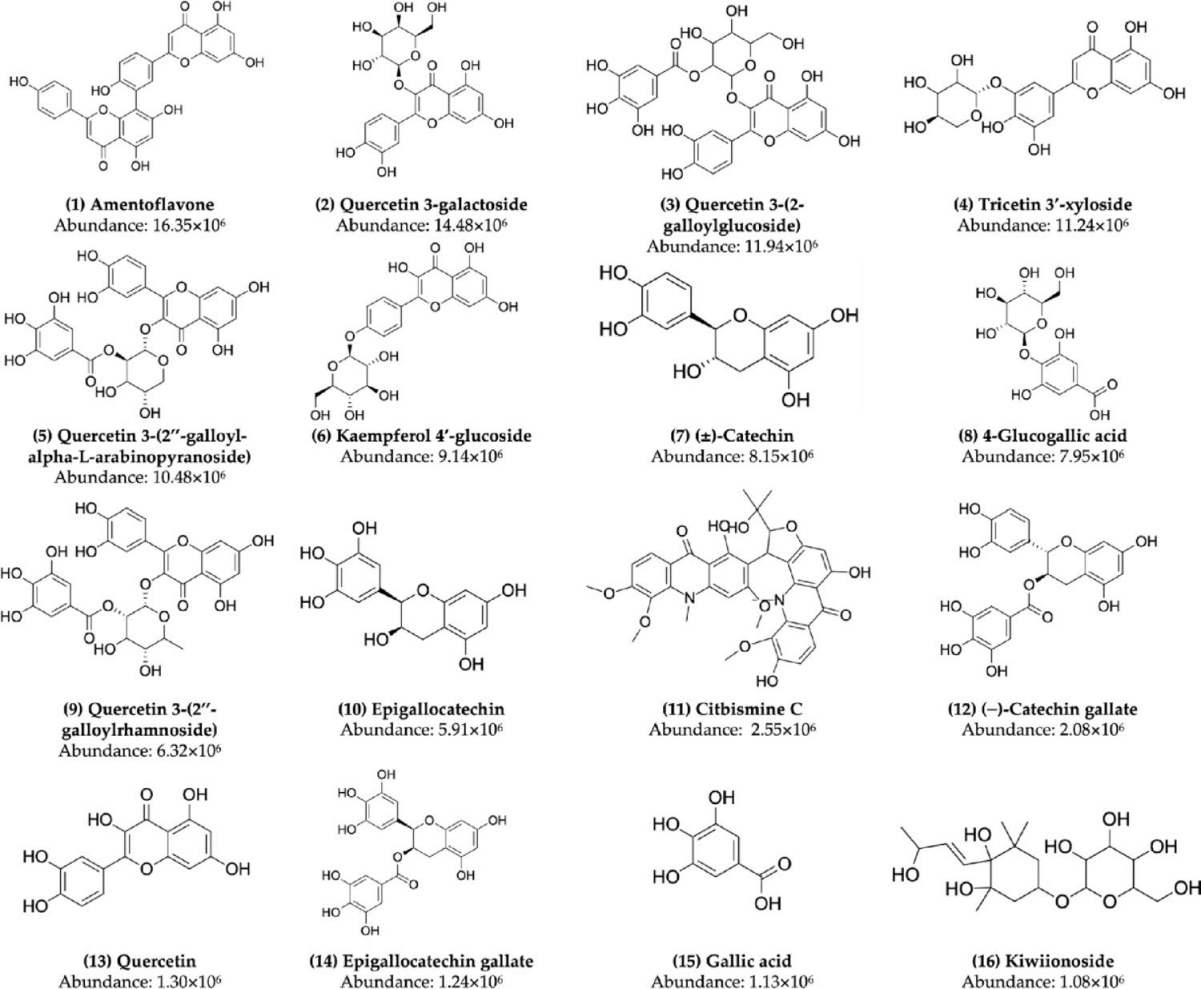
Cashew leaf phytochemicals selected for this structure-based virtual screening. Ligands are ordered according to relative abundance from the characterization study of Sinlapapanya et al. (2022)

The two-dimensional representation of the compounds from cashew leaves (Fig. 2), obtained from the study by Sinlapapanya et al. (2022), was plotted and rendered using the software MarvinSketch^®^ (https://chemaxon.com/marvin). In this step, the tool was configured to apply a structural optimization with very strict settings using the Merck Molecular Force Field (MMFF94) method (Halgren 1996), ensuring the selection of the conformer with the lowest energy.

### Molecular lipophilicity potential (MLP) and structural complexity

Subsequently, an analysis of the molecular lipophilicity potential (MLP) was performed, as presented in the following formula:

$$MLP=\sum_{i=1}^{N}{F}_{i}f\left({d}_{ik}\right)$$

Where *N* represents the number of molecular fragments (*i*) in the respective lipophilicity indices (*F*), and *f*(*d*) corresponds to the spatial distance function between two fragments *i* and *k* (Oberhauser et al. 2014). The obtained results were associated with the physicochemical descriptors of intrinsic lipophilicity (logP) and topological polar surface area (TPSA) (Nunes da Rocha et al. 2023). Furthermore, the compounds were categorized according to the relationship between lipophilicity and polarity, as described in the rule proposed by Pfizer, Inc. (Hughes et al. 2008).

Next, a quantitative estimation of structural complexity was performed using the MCE18 algorithm. The following formula was used to make the quantitative estimate of druglikeness:

$$\begin{aligned}MCE18&=\bigg(AR+NAR+Chiral\\ &\quad +Spiro\frac{Fsp3+Cyc-Acyc}{1+Fsp3}\bigg)Q^1\end{aligned}$$

Where, AR number of aromatic rings; NAR number of non-aromatic rings; Fsp3 fraction of sp3 atoms; Cyc cyclic structure; Acyc acyclic structure.

These parameters were evaluated based on current trends in medicinal chemistry, which emphasize the selection of larger and more polar compounds compared to those associated with intracellular toxicity. For the prioritization of candidate compounds, the results were categorized according to the following criteria:

(i) MCE18 below 63, indicating low three-dimensional complexity and traditional or widely known chemical structures;
(ii) MCE18 in the range of 63 to 100, characterizing compounds with high similarity to molecules described in patents, maintaining an appropriate balance between structural complexity and synthetic feasibility;
(iii) MCE18 above 100, representing highly complex structures, yet with limited synthetic accessibility (Ivanenkov et al. 2019).

The preference for MCE18 over Lipinski’s rule of five places greater emphasis on the cumulative sp3 score, a 3D drug-like property. In contrast, Lipinski’s rule is primarily concerned with two-dimensional topological properties. MCE18 performs a quantitative drug-likeness assessment based on the distribution of Fsp3 between AR and NAR structures. The presence of chiral and spirocyclic centers can result in conformational variations that are determinants for the route of administration and complexation on more selective biological targets. These variations generally violate the rule of five (Ivanenkov et al. 2019).

### PAMPA-based ADME prediction

For corroboration, the absorption, distribution, metabolism, and excretion (ADME) properties expressed in Parallel Artificial Membrane Permeability Assay (PAMPA) descriptors were predicted using a consensus test between the ADMETlab 3.0 (https://admetlab3.scbdd.com/) and ADMET-AI (https://admet.ai.greenstonebio.com/) databases. The evaluated properties included passive cellular permeability (*P*_app_) in colorectal adenocarcinoma (Caco-2) and Madin-Darby Canine Kidney (MDCK) cells, P-glycoprotein (P-gp) substrate status, intrinsic hepatic clearance (*Cl*_int, u_), clearance in the human liver microsome system (*Cl*_Micro_), clearance in human hepatocytes (*Cl*_Hepa_), and steady-state volume of distribution (*V*_dss_) (Da Rocha et al. 2024a, 2024b; Nunes et al. 2024).

### Site of metabolism and acute rat toxicity

For metabolic stability prediction, a site of metabolism and acute toxicity prediction in rats was performed. The XenoSite (https://xenosite.org/) and STopTox (https://stoptox.mml.unc.edu/) servers were configured to predict sensitivity to reactivity via metabolic activation, dependent on cytochrome P450 (CYP450) isoforms. Subsequently, the results were correlated with lethal dose (LD_50_) descriptors in rats for oral, intraperitoneal (IP), intravenous (IV), and subcutaneous (SC) administration routes, using the GUSAR Online – Way2Drug server (https://www.way2drug.com/gusar/) (Lima et al. 2023).

### Ecotoxicity prediction

The compounds, represented by their SMILES codes, were analyzed for ecotoxicity through a systematic approach using the ADMETlab 3.0 tool (https://admetlab3.scbdd.com/). The prediction covered both organic and exposure toxicity endpoints, including the following parameters: bioconcentration factor (BCF); growth inhibition (IGC_50_) in the aquatic protozoan species *Tetrahymena pyriformis*; lethal concentration (LC_50_) in fathead minnow (96 h) and *Daphnia magna* (48 h); organ toxicity, considering cardiotoxicity, ototoxicity, skin sensitization, carcinogenic potential, Ames mutagenicity, rat acute toxicity (ROA) and human hepatotoxicity (H-HT); as well as exposure toxicity, assessed based on eye corrosion (EC), eye irritation (EI), and neurotoxicity. The predicted probability values were processed using the Morpheus statistical tool (https://software.broadinstitute.org/morpheus/), enabling the generation of graphical representations in the form of heatmaps (Lopes et al. 2025).

Furthermore, a prediction of disruption in the human endocrine system was made to predict toxicity from exposure to chemical agents. This analysis was conducted using nuclear receptor modeling from the Tox21 dataset, including Aryl Hydrocarbon Receptor (AhR), Androgen Receptor (AR), Androgen Receptor Ligand Binding Domain (AR-LBD), Aromatase, Estrogen Receptor Alpha (ER), Estrogen Receptor Ligand Binding Domain (ER-LBD), and Peroxisome Proliferator-Activated Receptor Gamma (PPAR-Gamma) (Attene-Ramos et al. 2013). The compounds were classified as either negative or active for toxic response, with probability values exceeding 0.7 indicating higher toxicity.

### Obtaining and Preparing enzymes for docking studies

To study the mechanism of action of the phytochemicals present in cashew leaves (*Anacardium occidentale*) against *Cryptococcus neoformans*, molecular docking simulations were performed using the enzymes farnesyltransferase (CnFTase), beta-carbonic anhydrase (β-CA), and adenylosuccinate synthetase (AdSS) as targets. These enzymes were obtained from the Protein Data Bank (https://www.rcsb.org/) with PDB IDs: 7T08 (Wang et al. 2022), 2W3N (Schlicker et al. 2009), and 5I34 (Blundell et al. 2016), respectively, in *Cryptococcus neoformans* and expressed in the *Escherichia coli* system. During the preparation of the targets, residues were removed, essential cofactors were maintained, and polar hydrogens and Kollman charges were added using the AutoDockTools™ software (https://autodocksuite.scripps.edu/adt/) (Morris et al. 2009; Yan et al. 2014).

The Ramachandran plot is a crucial tool for assessing the quality and reliability of protein structures. It provides insights into the stereochemical quality of the protein by plotting the backbone dihedral angles φ and ψ of amino acid residues (Asrar et al. 2025). The validation of the structure of CnFTase, β-CA and AdSS was conducted using Ramachandran plot. To achieve this, the SAVES (saves.mbi.ucla.edu), an online server from the UCLA-DOE Institute for Genomics and Proteomics, was utilized, which offers interactive tools to validate the quality and accuracy of protein structures. Additionally, the prediction of possible binding sites in the enzymes (cavities) was carried out using the ProteinsPlus virtual platform (https://proteins.plus/), and the sites were defined within the parameters of volume, surface, and depth.

### Molecular docking simulation and data output

Molecular docking simulations were performed using the AutoDockVina software, employing the Lamarckian Genetic Algorithm (LGA), with an exhaustiveness of 64 and a grid box covering the entire structure of the protein targets. The grid dimensions were as follows: for CnFTase, the axes 26.268 x, -32.729 y, -3.094 z and size 102 x, 122 y, 100 z; for β-CA, the axes 29.330 x, 16.426 y, 13.510 z and size 122 x, 80 y, 98 z; and for AdSS, the axes 21.973 x, 45.740 y, 27.111 z and size 82 x, 102 y, 112 z. For comparative data, simulations were also performed with Amphotericin B and Fluconazole. Fifty simulations were run, generating 20 poses per simulation. The best pose selection criterion was based on the statistical parameter Root Mean Square Deviation (RMSD) with values up to 2.0 Å and an affinity energy (*E*_A_) lower than − 6.0 kcal/mol (Marinho et al. 2020).

### NMA-based MD simulation

Normal mode analysis (NMA) based on an elastic network model (ENM) was conducted to evaluate the conformational stability of the of the CnFTase receptor (PDB ID: 7T08) in complex with the lead compounds with best pharmacokinetic profile, and best molecular docking interaction, kaempferol 4’-glucoside, tricetin 3’-xyloside, and fluconazole (control); and for AdSS receptor (5I34) in complex with quercetin 3-galactoside and fluconazole (control) using the iMODS web server (https://imods.iqf.csic.es/), to assess the structural flexibility and stability of the receptor-ligand complex obtained in the molecular docking simulations. The PDB complex was loaded into iMODS, where NMA was performed to evaluate the conformational MD of the complex. The stability of the complex is indicated by the relationships between RMSD Cα and iteration index (López-Blanco et al. 2014; da Rocha et al. 2024b).

## Results and discussion

### Molecular lipophilicity potential (MLP) and structural complexity

A new physicochemical trend has been observed in drugs patented in recent years. This novel chemical uniqueness focuses on the selection of compounds that are more polar and more lipophilic than those typically approved by classic benchmark rules, such as the Lipinski rule, which indicates that a drug has good oral bioavailability when its molecular weight (MW) is < 500 g/mol and logP < 5 (Ivanenkov et al. 2019; Lipinski 2004). Furthermore, this emerging structural trend aims to understand how the structural complexity arising from the Fsp3, distributed between cyclic and acyclic structures, can affect pharmacokinetic attributes and the synthetic accessibility of new drugs (Ivanenkov et al. 2019). This shift in drug design reflects a deeper understanding of how modifying the balance between lipophilicity and polarity affects therapeutic efficacy and the synthetic accessibility of new drugs, aligning with current trends in medicinal chemistry focused on optimizing both bioavailability and manufacturability.

This approach, designated as the MCE18 criterion, employs a quantitative estimation mechanism for druglikeness based on multiparametric optimization. This mechanism involves the analysis of the weight factor of each physicochemical property, in addition to estimating the degree of desirability in relation to the ideal threshold. In contrast to conventional rules, such as Lipinski’s Rule, which have a tendency to eliminate drug candidates with a classificatory character, the MCE18 criterion does not disregard the multifactorial nature of the alignment between physicochemical attributes. This approach has the potential to identify promising molecules that may have been overlooked by conventional methods.

When the ligands were classified according to druglikeness scores, it was observed that the compounds (2) quercetin 3-galactoside, (4) tricetin 3’-xyloside, (6) kaempferol 4’-glucoside, (12) (-)-catechin gallate, and (14) epigallocatechin gallate fall within the range of 63 < MCE18 ≤ 100, indicating an appropriate degree of structural complexity that reflects similarity to compounds registered in patents in recent years. Meanwhile, compounds with trivial structures, i.e., those with MCE18 scores < 63, were excluded from the screening (Table 1) (Ivanenkov et al. 2019). At this threshold, compounds with MCE18 > 88.6 demonstrate a strategic increase in Fsp3 > 0.2, within the range of MW < 500 g/mol and logP < 1.0, due to replacement by scaffolds with higher saturation, as a strategy to optimize aqueous solubility and cell permeability (Wei et al. 2020). It is noteworthy that any compound with systemic distribution (in blood plasma) must inherently exhibit enhanced solubility. One of the strategies employed to optimize aqueous solubility involves reducing aromaticity (Lovering et al. 2009). It has been demonstrated that the outcomes align with the prevailing trend, as the diminution of Fsp3 within the MW thresholds has been observed to result in a reduction of logP for compounds (2) quercetin 3-galactoside, (4) tricetin 3’-xyloside, and (6) kaempferol 4’-glucoside, with rates falling below 0.85 (Table 1).

Additionally, the compounds with MCE18 > 100, such as the derivatives (3) quercetin 3-(2 galloylglucoside), (5) quercetin 3-(2’’-galloyl-alpha-L-arabinopyranoside), (9) quercetin 3-(2’’-galloylrhamnoside), and (11) citbismine C, exhibited greater structural complexity, which may reduce their synthetic accessibility and pharmacokinetic viability (Table 1).

**Table 1:** Physicochemical properties calculated and applied to the medicinal chemistry scoring criteria by MCE18 of phytochemicals from cashew leaves

Compound	logP	MW (g/mol)	TPSA (Å^2^)	Fsp3	Flex	SC	MCE18
Amentoflavone	6.00	538.09	181.8	0.00	0.08	0	36.00
**Quercetin 3-galactoside**	**0.46**	**464.10**	**210.51**	**0.29**	**0.17**	**5**	**91.00**
Quercetin 3-(2 galloylglucoside)	1.08	616.11	277.27	0.21	0.23	5	115.29
**Tricetin 3’-xyloside**	**0.81**	**434.08**	**190.28**	**0.25**	**0.13**	**4**	**88.40**
Quercetin 3-(2’’-galloyl-alpha-L-arabinopyranoside)	1.72	586.10	257.04	0.19	0.19	4	112.35
**Kaempferol 4’-glucoside**	**0.69**	**448.10**	**190.28**	**0.29**	**0.17**	**5**	**87.63**
(±)-Catechin	1.34	290.08	110.38	0.20	0.06	2	60.00
4-Glucogallic acid	–0.79	332.07	177.14	0.46	0.31	5	56.42
Quercetin 3-(2’’-galloylrhamnoside)	2.22	600.11	257.04	0.21	0.19	5	115.29
Epigallocatechin	0.78	306.07	130.61	0.20	0.06	2	63.33
Citbismine C	4.49	684.23	171.07	0.30	0.16	2	148.00
**(−)-Catechin gallate**	**2.25**	**442.09**	**177.14**	**0.14**	**0.17**	**2**	**84.24**
Quercetin	2.16	302.04	131.36	0.00	0.06	0	19.00
**Epigallocatechin Gallate**	**1.89**	**458.08**	**197.37**	**0.14**	**0.17**	**2**	**87.48**
Gallic acid	0.65	170.02	97.99	0.00	0.14	0	8.00
Kiwiionoside	–0.95	406.22	160.07	0.90	0.39	9	57.44

Lead compounds filtered by MCE18 are highlighted in bold

**Fig. 3:**
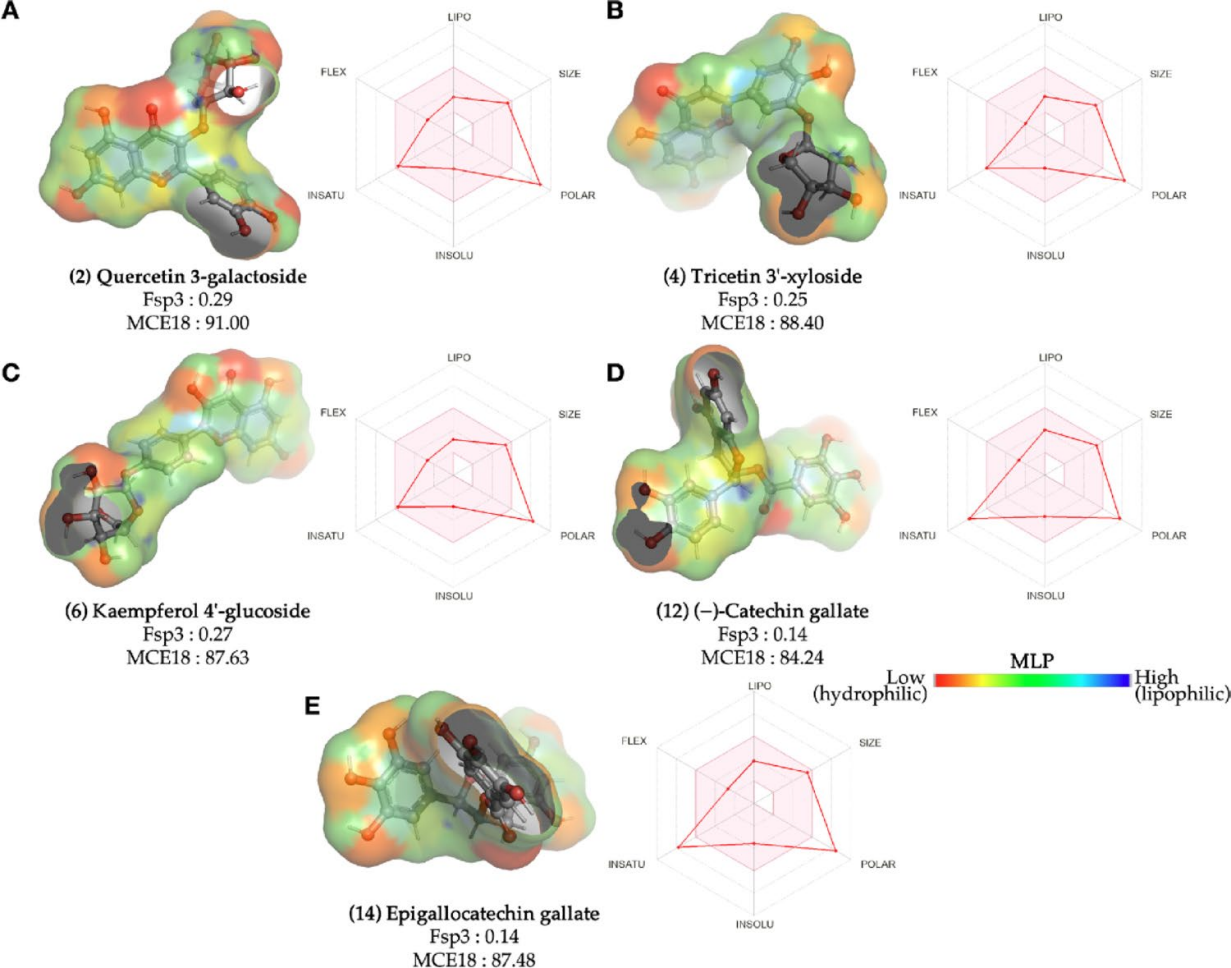
Surface map of molecular lipophilicity potential (MLP) and medicinal chemistry desirability radar, respectively, of the lead compounds screened by Medicinal Chemistry Evolution, 2018 (MCE18): **a** quercetin 3-galactoside, **b** tricetin 3´-xyloside, **c** kaempferol 4´-glucoside, **d** (-)-ctachin gallate and **e** epigallocatechin gallate

An analysis of the optimized chemical structures of the compounds reveals that those with optimized MCE18 exhibit well-defined aromatic structures on their hydrophobic surfaces (yellow to green spectra), as depicted in the MLP map (Fig. 3). Compound (2), quercetin 3-galactoside, possesses a polar surface area (TPSA) of approximately 210.51 Å^2^, with a notable contribution from the four hydroxyl groups (-OH) substituted on its aromatic rings and the 4’-glucoside substructure (red color spectra) (Ertl 2007), resulting in a hydrophilic surface on its oxygenated groups (Fig. 3a). Derivatives (4) tricetin 3’-xyloside (Fig. 3b) and (6) kaempferol 4’-glucoside (Fig. 3c) have the same topological polarity (TPSA = 190.28 Å^2^), as they have the same number of polar groups, where the glycoside group contributes strongly to the formation of hydrophilic surfaces (red color spectra). Furthermore, these molecules exhibit a minimum of 0.25 of their molecular fraction in a hybridized sp3 (Fsp3) configuration, as delineated in Table 1. This observation places them within the instauration spectrum for novel structural trends that ensure optimal synthetic accessibility.

The lead compounds, i.e., the derivatives (2) quercetin 3-galactoside, (4) tricetin 3’-xyloside, and (6) kaempferol 4’-glucoside, are within the ideal thresholds for molecular weight and unsaturation (at the same time), as observed by the bioavailability radar in Fig. 3. MPL surface analysis indicates that the majority of the hydrophilic surface is concentrated in the glycosidic substructure, which is rich in C_sp3_-H groups (low MLP – red color spectra). This optimization of properties leads to enhanced aqueous solubility (INSOLU) and lipophilicity (Fig. 3), aligning with the trends predicted by structural complexity analysis (Lovering et al. 2009).

**Fig. 4:**
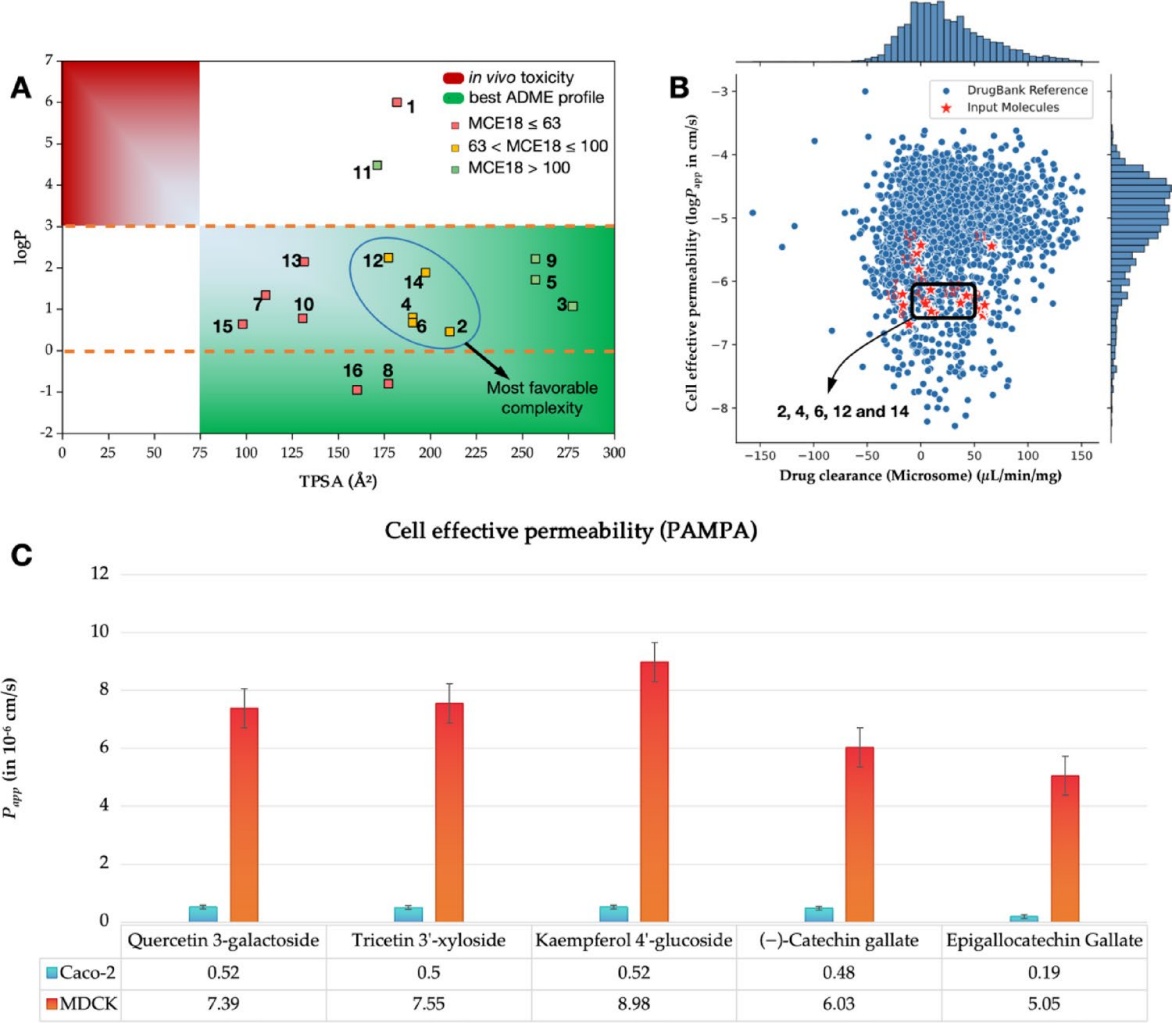
**a** Alignment between lipophilicity (logP) and polarity, by the topological polar surface area (TPSA) descriptor, to estimate the desirability spectrum, where the compounds were classified by their MCE18 scores. **b** structural similarity test with compounds deposited in the database for the cell effective permeability (*P*_app_) and drug clearance in microsome descriptors, guided by artificial intelligence, and **c** *P*_app_ prediction in colorectal adenocarcinoma (Caco-2) and Madin-Darby Canine Kidney (MDCK) cell lines for the lead compounds identified by MCE18

The medicinal chemistry desirability radar (Fig. 3) indicates that the compounds (2) quercetin 3-galactoside (Fig. 3a), (4) tricetin 3’-xyloside (Fig. 3b), and (6) kaempferol 4’-glucoside (Fig. 3c) may possess a higher degree of polarity compared to certain compounds commonly identified as oral drug candidates (TPSA > 140 Å^2^). When the polarity descriptors (TPSA) were aligned with the lipophilicity descriptors (logP), it was observed that the compounds, with the exception of the derivatives (1) amentoflavone and (11) Citbismine C, reside in a physicochemical space formed by compounds with low lipophilicity and high polarity (logP < 3 and TPSA > 75 Å^2^), indicating a low incidence of toxicity in vivo. Of particular interest is the observation that the MCE18 values exhibited a tendency to increase as the polarity of the compounds increased. This tendency is exemplified by the derivatives (2) quercetin 3-galactoside, (4) tricetin 3’-xyloside, (6) kaempferol 4’-glucoside, (12) (-)-catechin gallate and (14) epigallocatechin gallate are highly polar (TPSA 175–225 Å^2^) and fall within the range of acceptable structural complexity of MCE18 between 63 and 100 (Fig. 4a).

### PAMPA-based ADME prediction

Wager et al. (2016) reported in their multiparametric optimization analysis that compounds more polar than drugs commonly approved as oral route drugs may present a moderate cell permeability (*P*_app_ in order of 10^− 6^ cm/s) and low intrinsic hepatic clearance (*Cl*_int, u_ < 8.0 mL/min/kg), due to metabolic stability (Pettersson et al. 2016; Wager et al. 2016). In a screening guided by artificial intelligence (AI) (Swanson et al. 2024), it was possible to observe a similarity between the compounds (2) quercetin 3-galactoside, (4) tricetin 3’-xyloside, (6) kaempferol 4’-glucoside, (12) (-)-catechin gallate, and (14) epigallocatechin gallate. These findings are consistent with compounds deposited in the Drugbank^®^ database, which exhibit low clearance in hepatic microsomes (*Cl*_Micro_ 0.0–50.0 µL/min/mg) and maintain an effective cell permeability range (log*P*_app_ close to -6.0 cm/s) (Fig. 4b).

In general, a profile compatible with drugs having a MW of less than 500 g/mol and a logP < 5 that exhibit metabolic stability is observed. This corroborates a favorable oral bioavailability profile. According to Johnson et al. (2009), small, poorly lipophilic compounds are metabolically less susceptible to the cytochrome P450 (CYP450) enzyme system in human liver microsomes (HLMs) (Johnson et al. 2009), where low hepatic clearance induces gradual release into blood plasma (*Cl*_int, u_: < 5.0 mL/min/kg) (Pettersson et al. 2016) (Table 2), ensuring good oral bioavailability, although experimental compounds from Pfizer exhibit TPSA > 100 Å², they have *P*_app_ MDCK values on the order of 10⁻⁶ cm/s. This indicates that permeability and bioavailability are multifactorial and depend on attributes such as lipophilicity (logP) and MW (Möbitz 2024).

Conversely, the ADME predictive services consensus test indicates that these lead compounds possess *P*_app_ Caco-2 values of less than 6.0 × 10^− 7^ cm/s, suggesting their potential for oral administration due to a gradual absorption in the human gastrointestinal tract, representing a kinetic pattern of permeation rate (Fig. 4c). However, the predicted values of *P*_app_ MDCK > 5.0 × 10^− 6^ cm/s suggest that the derivatives are more permeable in cell lines with passive efflux mechanisms different from Caco-2, estimating a PAMPA profile based on the high cell viability for these compounds (Fig. 4c) (Ma et al. 2005). It is crucial to acknowledge that oral bioavailability is a multifactorial parameter influenced by intestinal residence time, solubility, and first-pass hepatic clearance (Lennernaäs 1998).

Among these, (2) quercetin 3-galactoside and (4) tricetin 3’-xyloside present a probability > 0.8 of being P-gp substrates, indicating that the higher permeability of MDCK compared to Caco-2 is due to the high susceptibility of the compounds to passive influx by intestinal epithelial cells, since the cell lines do not share the same transport and efflux mechanisms in their membranes (Table 2) (Jin et al. 2014).

**Table 2:** Pharmacokinetic descriptors predicted by consensus prediction of ADME properties for cashew leaf phytochemicals

Compound	*P*_app_ Caco-2 (cm/s)	*P*_app_ MDCK (cm/s)	*P*-gp efflux	Cl_int, u_ (mL/min/kg)	Cl_Micro_ (µL/min/mg)	Cl_Hepa_ (µL/min/10^6^ cells)	V_dss_ (L/kg)
Amentoflavone	5.46 × 10^− 6^	8.00 × 10^− 6^	0.02	3.82	-3.60	2.30	0.48
**Quercetin 3-galactoside**	**5.19 × 10** ^**− 7**^	**7.40 × 10** ^**− 6**^	**0.88**	**4.86**	**12.97**	**30.42**	**0.88**
Quercetin 3-(2 galloylglucoside)	3.09 × 10^− 7^	7.02 × 10^− 6^	0.02	6.76	57.78	24.39	0.67
**Tricetin 3’-xyloside**	**5.01 × 10** ^**− 7**^	**7.55 × 10** ^**− 6**^	**0.99**	**3.59**	**4.43**	**31.17**	**0.90**
Quercetin 3-(2’’-galloyl-alpha-L-arabinopyranoside)	4.18 × 10^− 7^	8.31 × 10^− 6^	0.02	7.82	55.88	27.53	0.66
**Kaempferol 4’-glucoside**	**5.25 × 10** ^**− 7**^	**8.98 × 10** ^**− 6**^	**0.41**	**2.51**	**9.72**	**35.47**	**0.91**
(±)-Catechin	8.87 × 10^− 7^	4.55 × 10^− 6^	0.01	17.30	-17.04	53.07	0.66
4-Glucogallic acid	3.39 × 10^− 7^	2.95 × 10^− 6^	0.21	1.74	-10.98	33.45	0.37
Quercetin 3-(2’’-galloylrhamnoside)	4.01 × 10^− 7^	9.67 × 10^− 6^	0.02	7.82	59.68	24.92	0.64
Epigallocatechin	3.38 × 10^− 7^	4.04 × 10^− 6^	0.01	14.94	-16.47	46.71	0.60
Citbismine C	3.01 × 10^− 6^	2.26 × 10^− 6^	0.96	1.29	66.12	19.01	0.36
**(−)-Catechin gallate**	**4.78 × 10** ^**− 7**^	**6.03 × 10** ^**− 6**^	**0.00**	**17.28**	**42.49**	**70.74**	**0.47**
Quercetin	6.25 × 10^− 6^	7.69 × 10^− 6^	0.00	8.28	0.08	75.31	0.58
**Epigallocatechin Gallate**	**1.92 × 10** ^**− 7**^	**5.05 × 10** ^**− 6**^	**0.00**	**14.45**	**37.11**	**61.76**	**0.51**
Gallic acid	1.87 × 10^− 6^	5.11 × 10^− 6^	0.00	10.11	-1.78	41.23	0.47
Kiwiionoside	1.61 × 10^− 6^	1.15 × 10^− 3^	0.36	1.38	9.09	40.78	0.43

Lead compounds filtered by MCE18 are highlighted in bold

### Site of metabolism and toxicity

The analysis of the metabolic site is imperative for the selection of safer compounds by metabolic activation, particularly among drug candidates. This reactivity is driven by the hepatic metabolism of the CYP450 isoenzyme system and can affect the oral bioavailability and daily oral dose administered of these new candidates (Yu et al. 2014; Zheng et al. 2009). In the context of rational drug planning, the prediction of metabolism sites aims to identify biotransformation sites that lead to the formation of reactive secondary metabolites, such as aromatic centers susceptible to epoxidation and dealkylation of nitrogen and oxygen groups (Dang et al. 2018; Hughes et al. 2015).

**Fig. 5:**
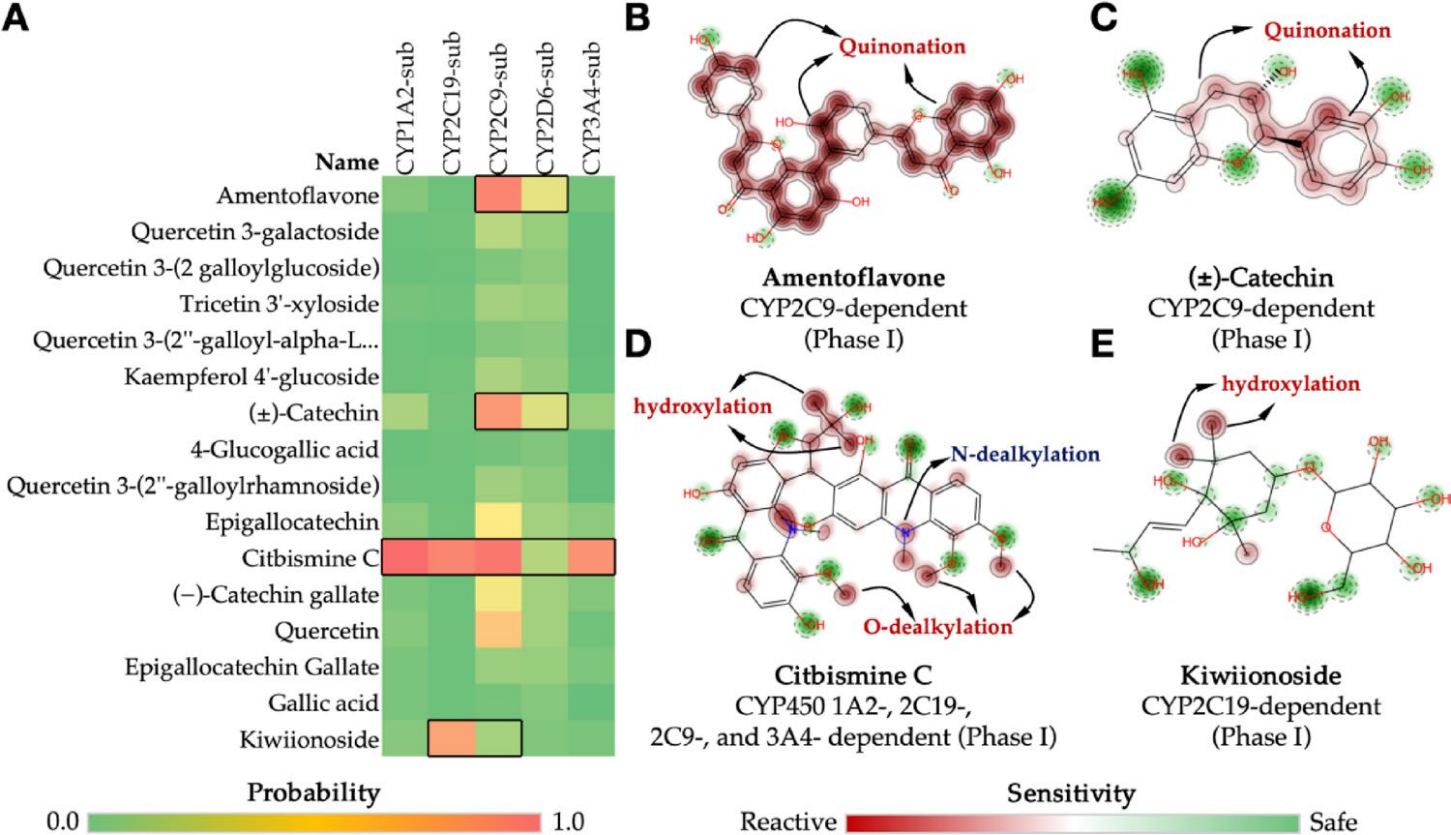
**a** Heatmap predicting the likelihood of cashew phytochemicals being substrates of CYP450 isoforms (1A2, 2C19, 2C9, 2D6 and 3A4), and predicting the site of metabolism for the compounds that showed reactivity for being CYP450 substrates: **b** amentoflavone, **c** (±)-catechin, **d** citbismice C and **e** kiwiionoside

A consensus prediction of ADME properties was conducted, which revealed that the compounds amentoflavone and (±)-catechin are substrates of the CYP2C9 isoform (Fig. 5a). Structural analysis reveals that the polyphenolic moieties of these compounds are susceptible to quinonation by this isoform, resulting in the formation of electrophilic receptors capable of conjugation to nucleophilic sites on DNA and proteins (Fig. 5b and c) (Hughes and Swamidass 2017). The chemical structure of citbismine C features a series of -OCH_3_ and -NCH_3_ groups, which are O- and N- dealkylation sites dependent on the CYP1A2, CYP2C19, CYP2C9, and CYP3A4 isoforms. Consequently, these groups form a series of secondary metabolites that can effectively reduce the metabolic stability and plasma distribution of the compound (Fig. 5d). Furthermore, kiwiionoside can undergo hydroxylation at its terminal methyl (-CH_3_) groups by the CYP2C9 isoform (Fig. 5e).

Conversely, the derivatives quercetin 3-galactoside, tricetin 3’-xyloside, kaempferol 4’-glucoside, (-)-catechin gallate, and epigallocatechin gallate, filtered as lead compounds by MCE18, exhibited a low probability of being substrates for the majority of CYP450 isoforms in the human liver, thereby ensuring greater metabolic stability for these compounds (Fig. 5a). Of these, the glycosylated derivatives exhibited greater metabolic stability in hepatic microsomes (*Cl*_Micro_ < 20 µL/min/mg) compared to the derivatives containing the gallate substructure (*Cl*_Micro_ > 30 µL/min/mg), a trend that extends to the levels of metabolic stability in cell hepatocytes, with *Cl*_Hepa_ values estimated at < 40 µL/min/10^6^ cells for the glycosylated derivatives and at > 60 µL/min/10^6^ cells for the gallate derivatives (Table 2) (Di et al. 2012).

As compounds of moderate lipophilicity, they may optimize plasma distribution for compounds with better metabolic stability, as indicated by *V*_dss_ values > 0.8 L/kg for the derivatives quercetin 3-galactoside, tricetin 3’-xyloside, and kaempferol 4’-glucoside. These findings suggest that these compounds may distribute more effectively in biological tissues than the other derivatives (Table 2) (Pires et al. 2018).

**Fig. 6:**
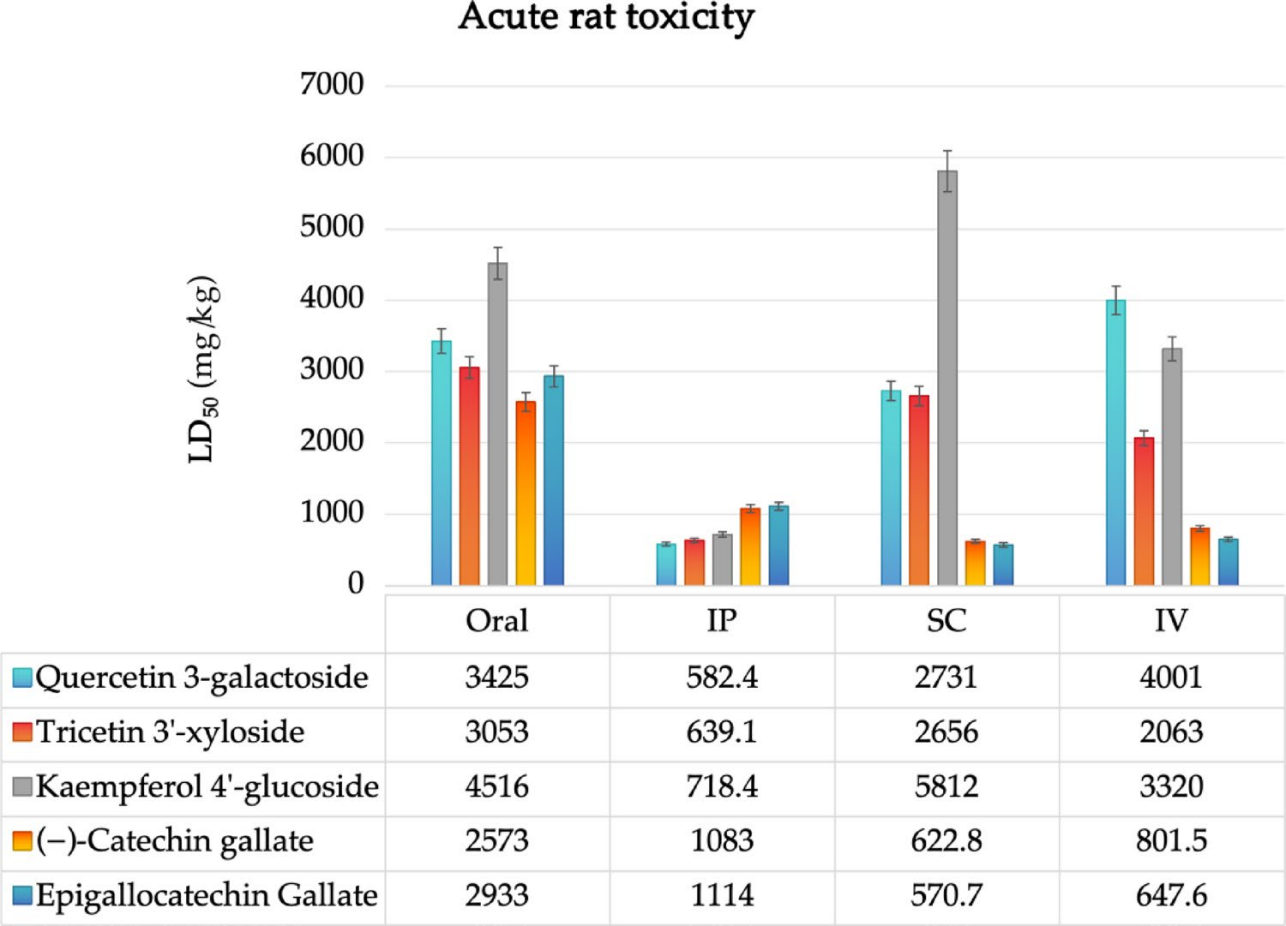
Prediction of acute toxicity in rats for different routes of administration: oral, intraperitoneal (IP), subcutaneous (SC) and intravenous (IV), considering the lead compounds filtered by MCE18

In the prediction of acute toxicity in rats, lethal dose (LD_50_) values greater than 3000 mg/kg were estimated for the glycosylated derivatives quercetin 3-galactoside, tricetin 3’-xyloside, and kaempferol 4’-glucoside. Of these, the kaempferol 4’-glucoside compound was particularly noteworthy, with an estimated LD_50_ of 4516 mg/kg (Fig. 6). This finding suggests that this class of compounds has a low probability of inducing a toxic response through metabolic activation when administered orally (Gonella Diaza et al. 2015). Furthermore, the compound demonstrated an estimated LD_50_ of 5812 mg/kg through subcutaneous (SC) administration, suggesting minimal toxicity potential following skin exposure (Fig. 6). Notably, another administration group exhibited LD_50_ values greater than 2000 mg/kg for the glycosylated derivatives quercetin 3-galactoside, tricetin 3’-xyloside, and kaempferol 4’-glucoside when administered intravenously (Fig. 6). This observation can be attributed to the metabolic stability of glycosides, which minimizes the likelihood of plasma bioconcentration of these compounds.

### Environmental ecotoxicity assessment

The ecotoxicity of chemical substances dispersed in the air and water can be directly related to the mortality of animal species in the impacted ecosystem. Determining the concentration capable of inducing lethality in a fish population within a specific period of 96 h can provide relevant data on the bioconcentration factor of low molecular mass organic compounds. This factor represents an essential parameter for assessing the distribution of these chemical substances between the atmosphere and the aquatic environment (Hayyan et al. 2024). As demonstrated in Fig. 6, the prediction results indicated that the lead compounds (quercetin 3-galactoside, tricetin 3’-xyloside, kaempferol 4’-glucoside, (-)-catechin gallate and epigallocatechin gallate) have the potential to trigger an acute toxic response in aquatic species, as evidenced by logLC_50_ values > 4.0 mg/L in Fathead Minnow and > 5.0 mg/L in the species *Daphnia magna*.

The results of the ecotoxicity prediction using environmental biomarkers showed a low bioconcentration factor (BCF), indicating a low probability of secondary intoxication by intracellular bioconcentration in human organisms via the food chain. From an environmental risk perspective, predicted LC_50_ values greater than 4.0, expressed in the unit -log_10_(mg/L)/(1000*MW), suggest that environmental exposure to these compounds could result in acute lethality of 50% in a population of the aquatic species *Pimephales promelas* (Fathead Minnow) after 96 h and *Daphnia magna* after 48 h (Fig. 6a). Conversely, the predicted IGC_50_ values > 3.0 (in -log_10_[mg/L]) indicates a risk of inhibiting the growth of the aquatic protozoan *Tetrahymena pyriformis*, suggesting that exposure to these compounds in aquatic ecosystems represents an environmental risk, affecting the microbiota responsible for environmental balance.

### Human toxicity and Pharmacological safety

**Fig. 7:**
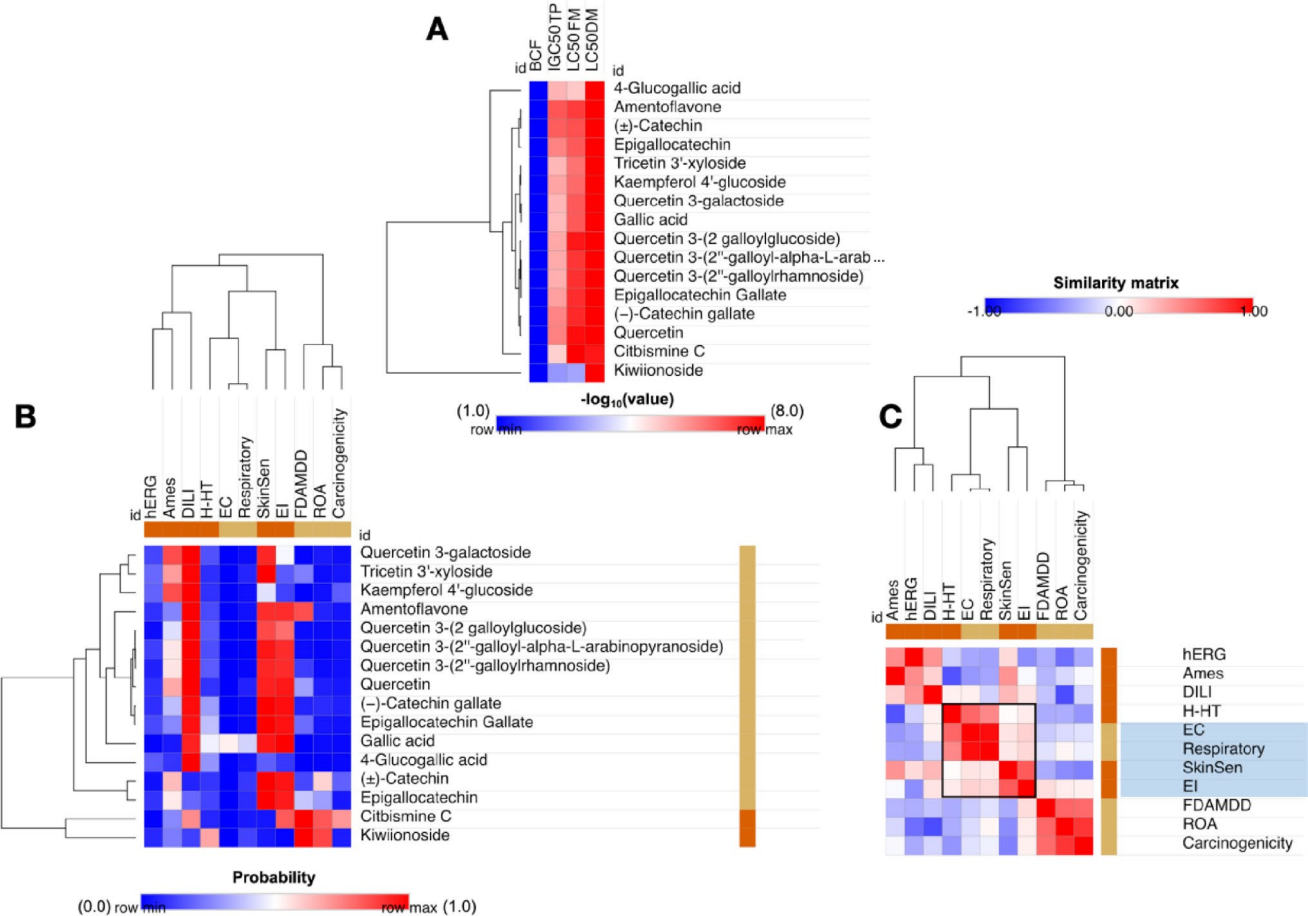
Systematic prediction of acute and organic toxicity and ecotoxicity, expressed in: **a** estimate of the bioconcentration factor (BCF), growth inhibition concentration (IGC_50_) of *Tetrahymena piriformis* (TP), inhibitory concentration (IC_50_) in Fathead Minnow (FM) and *Daphnia magna* (DM); **b** descriptors of organic toxicity and ecotoxicity; and **c** Pearson similarity matrix for the descriptors of environmental toxicity by exposure

In contrast, regarding human toxicity, the prediction suggests that all the compounds have a low incidence of EC and respiratory problems (Fig. 7b), as well as showing no tendency to inhibit the human Ether-a-go-go-Related Gene (hERG) channels, responsible for the flow of K+ ions in the cardiorespiratory system, indicating a low tendency to induce cardiotoxic responses such as cardiac arrhythmias (Radchenko et al. 2017).

However, a risk of contact toxicity, such as EI and skin sensitization, is estimated, highlighting the safety of exposure to these biomarkers in relation to the compound Kaempferol 4’-glucoside (blue color in the heatmap of Fig. 7b). Pearson’s correlation matrix showed a strong relationship between the descriptors of EC and respiratory toxicity (low probability) and skin sensitization and EI (high probability).

The results obtained indicate that, from a structural point of view, these substances are derivatives with the potential to induce irritability and certain toxic contact responses. This contributes to more accurate modeling in predicting toxicity resulting from exposure.

**Table 3:** Descriptors of toxicity by bioconcentration and acute toxicity in aquatic species, as biomarkers of ecotoxicity

Compound	BCF (L/kg)	IGC_50_ TP (-log_10_[mg/L])	LC_50_ FM (-log_10_[mg/L])	LC_50_ DM (-log_10_[mg/L])
Amentoflavone	0.71	5.19	5.47	6.11
Quercetin 3-galactoside	0.90	3.93	4.79	5.62
Quercetin 3-(2 galloylglucoside)	0.99	4.06	5.36	5.57
Tricetin 3’-xyloside	0.80	3.99	4.66	5.78
Quercetin 3-(2’’-galloyl-alpha-L-arabinopyranoside)	0.99	4.19	5.29	5.79
Kaempferol 4’-glucoside	0.79	3.86	4.38	5.34
(±)-Catechin	0.93	4.33	4.45	5.10
4-Glucogallic acid	0.36	1.93	1.84	2.79
Quercetin 3-(2’’-galloylrhamnoside)	0.98	4.21	5.44	5.90
Epigallocatechin	0.92	4.06	4.40	5.13
Citbismine C	0.68	4.68	7.43	7.14
(−)-Catechin gallate	0.95	4.44	5.29	5.67
Quercetin	1.02	4.23	5.22	5.33
Epigallocatechin Gallate	0.99	4.20	5.26	5.67
Gallic acid	0.34	2.60	3.26	3.89
Kiwiionoside	0.30	1.46	1.57	4.24

In addition, a prediction of endocrine disruption was conducted, with the objective of identifying compounds exhibiting antagonistic properties capable of modulating the activity of nuclear receptors (NRs). These compounds are designed to interfere with the functions of the endocrine system and hormonal processes. Consequently, strategies based on structure-activity relationships (SARs) are pertinent tools for correlating the chemical nature of a substance - including molecular weight, hydrophobicity, and topological properties - with its adverse effects on the endocrine system (Schneider et al. 2019).

**Fig. 8:**
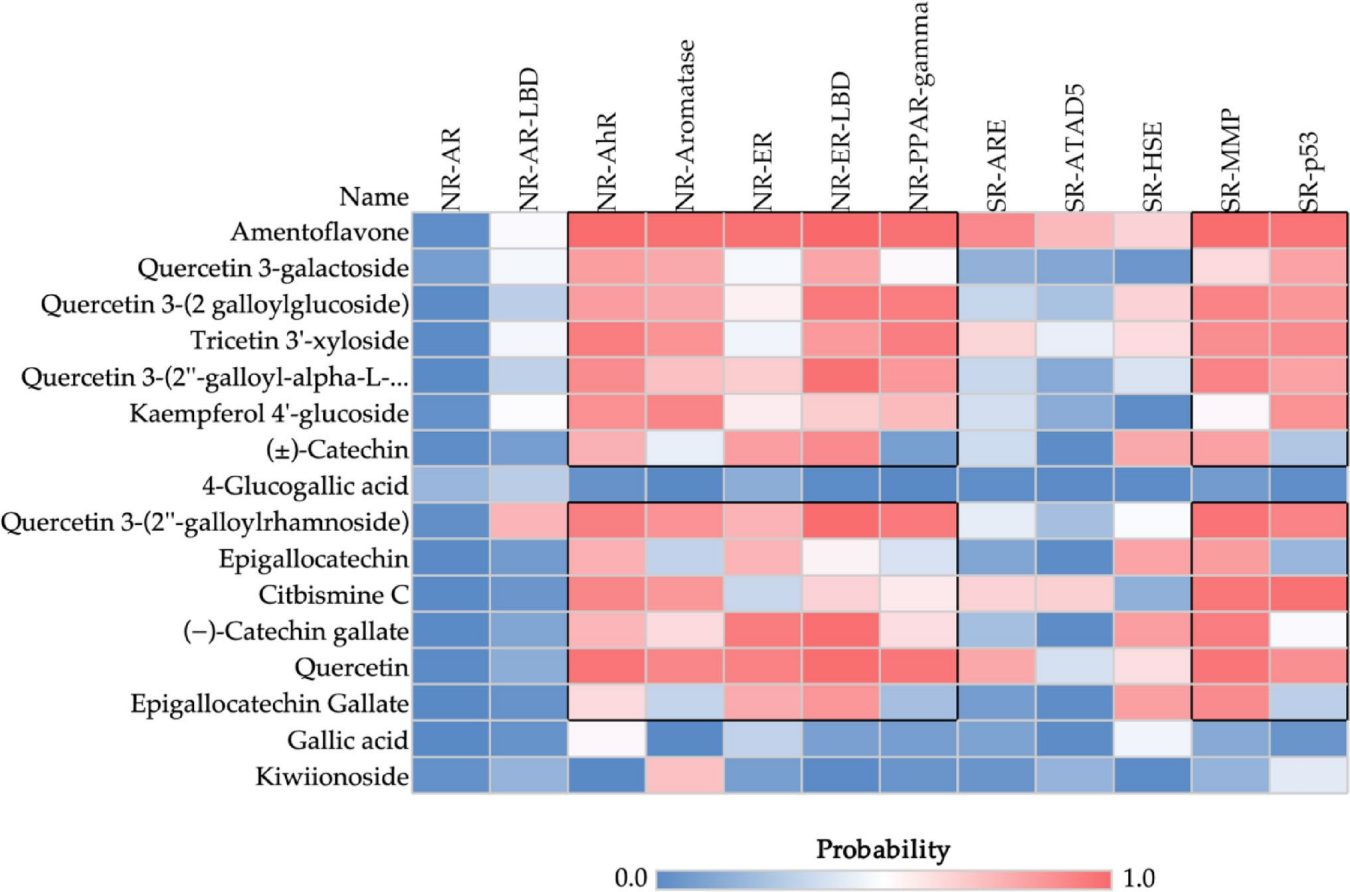
Probability heatmap for prediction of endocrine disruption toxicity. The predictive test is based on the similarity test with other nuclear receptor (NR) antagonists from the Tox21 database

As illustrated in Fig. 8, the heat map ranges from blue (indicating safety) to red (indicating toxicity), with the intensity of the color representing the prediction probability for nuclear receptors potentially affected by chemicals in the Tox21 dataset (Attene-Ramos et al. 2013). The results demonstrated that the compounds amentoflavone and quercetin can elicit the highest number of antagonistic responses in NRs, with probability > 0.7 (Fig. 8). Among the NRs exhibiting the most antagonistic interference within the prediction are aryl hydrocarbon receptors (AhR), aromatase, estrogen receptor ligand binding domain (ER-LBD), and peroxisome proliferator-activated receptor gamma (PPAR-gamma). The compounds 4-glucogallic acid, gallic acid, and kiwiionoside are the derivatives with the greatest safety against these models (Fig. 8). This observation indicates a substantial possibility that these compounds could negatively modulate the activity of these receptors, thereby interfering with human hormonal and metabolic processes that are mediated by them.

### Molecular Docking simulations

#### Validation of the target model

**Fig. 9:**
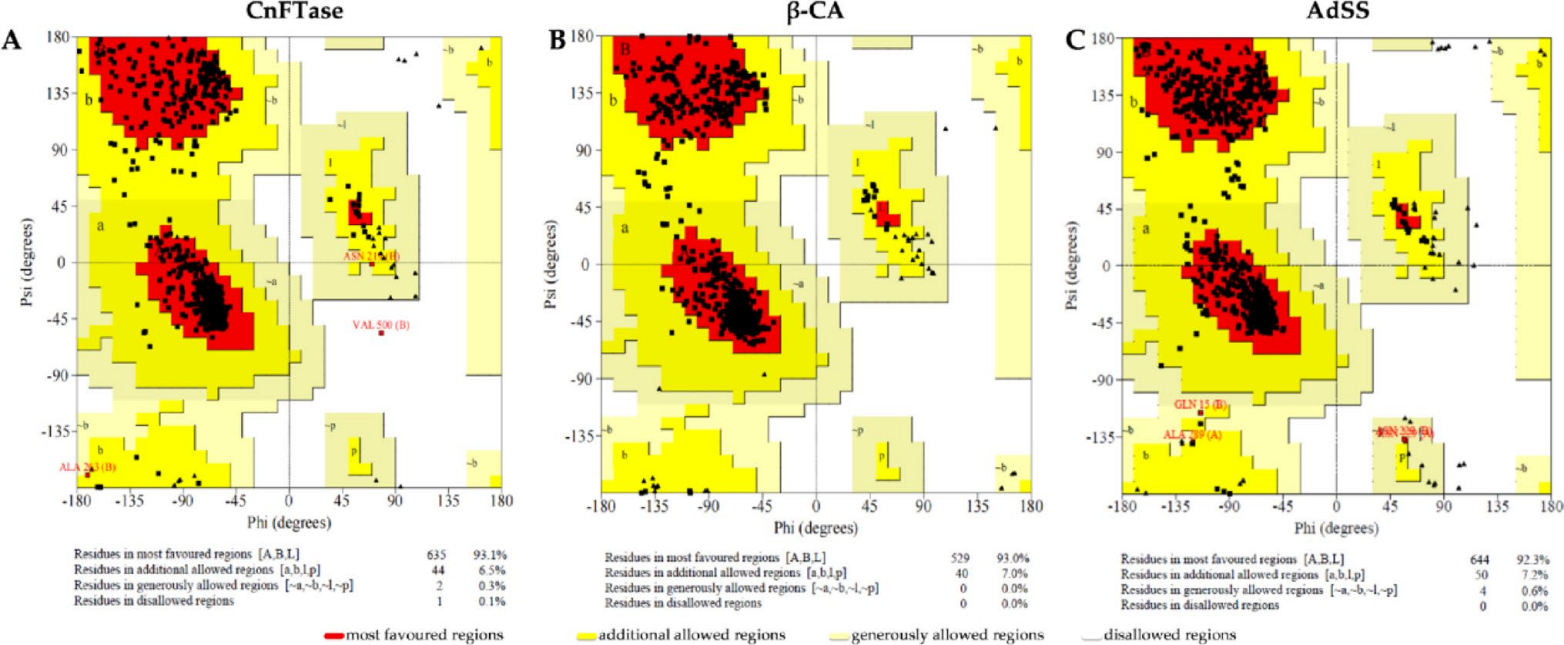
Target quality and Ramachandran plot. CnFTase (**A**), β-CA (**B**) and AdSS (**C**)

The validation of the target enzymes utilized was determined using the Ramachandran plot, which relates the degree of torsion of the φ (phi) and ψ (psi) angles formed between the main chain of the amino acid residues and the steric hindrance formed by their side chains to estimate occupancy in favorable regions. An occupancy value greater than 90% in the most favorable region indicates a reliable PDB target for pharmacodynamic simulations (Laskowski et al. 1993a; Laskowski et al. 1993b). In this context, the Ramachandran plots of CnFTase (Fig. 9a), β-CA (Fig. 9b), and AdSS (Fig. 9c) exhibited, respectively, 93.1%, 93.0%, and 92.3% of the residues in more favorable regions (red color spectra in Fig. 9), suggesting the reliability of the targets. However, it is noteworthy that CnFTase (Fig. 9a) exhibited a single amino acid residue (0.1%) in an impermissible region of the Ramachandran plot. This residue (Val-500), however, does not overlap with the cavity binding pocket predicted in Fig. 13a and b. Consequently, the stereochemistry and reliability of the enzymes can be validated.

### Binding affinity of the cashew leaves phytochemicals

In pursuit of alternative treatment options for cryptococcosis, we have conducted computer simulations employing the molecular docking technique to investigate the mechanism of action of cashew leaf phytochemicals against farnesyltransferase (CnFTase), β-carbonic anhydrase (β-CA), and adenylosuccinate synthetase (AdSS). The molecular docking study demonstrated that the compounds exhibited RMSD values within the parameter described in the literature of up to 2.0 Å (Shityakov and Foerster 2014; Yusuf et al. 2008) (Supplementary Tables S1, S2, and S3), thereby confirming the feasibility of forming receptor-ligand complexes. The data also exhibited favorable affinity energies for the receptor-ligand complexes formed.

**Fig. 10:**
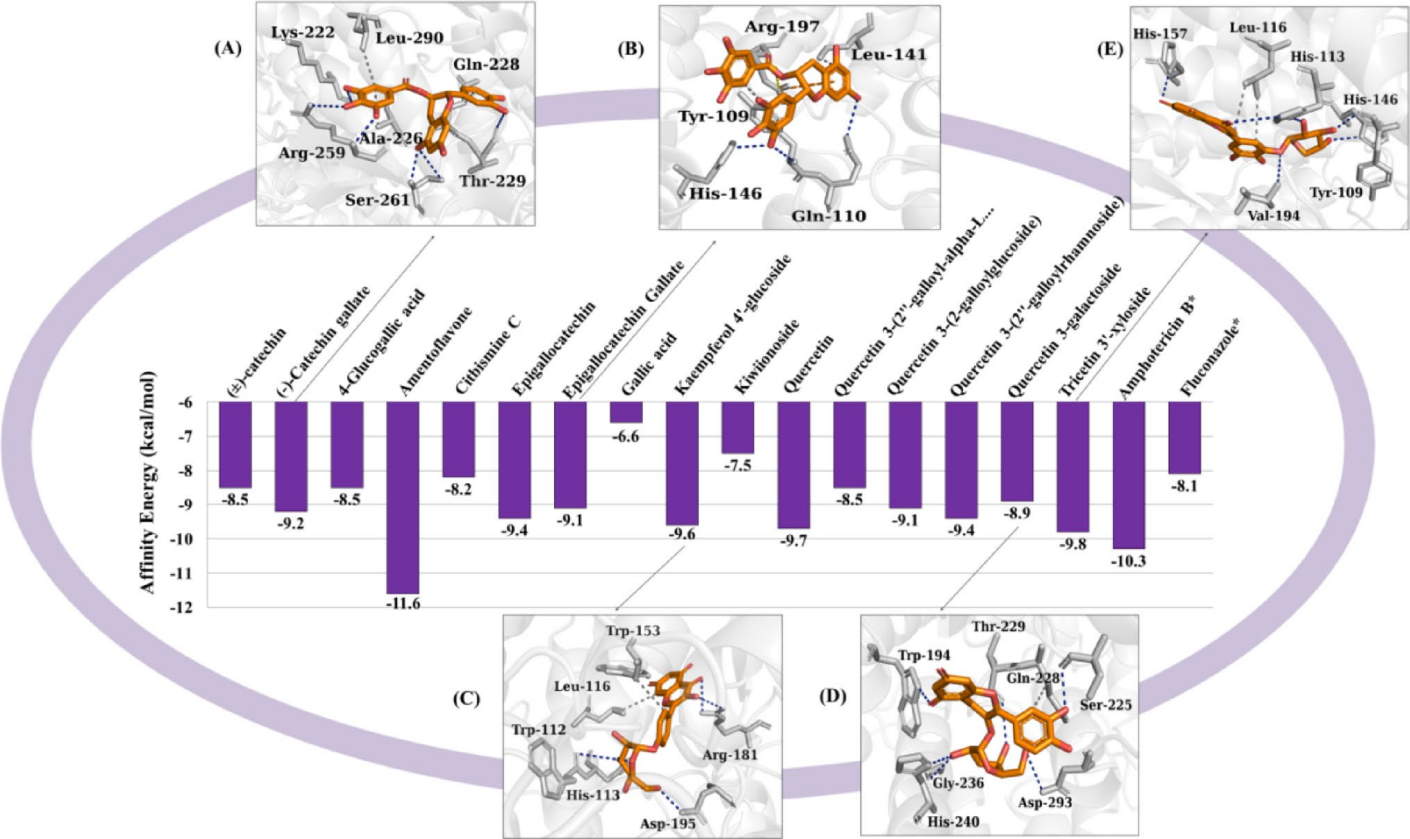
Affinity energy (kcal/mol) of the compounds against CnFTase

For the CnFTase enzyme, the following observations were made: (1) amentoflavone exhibited an affinity energy of -11.6 kcal/mol (Fig. 10); however, its pharmacokinetic viability is low. Therefore, the five compounds that demonstrated high pharmacokinetic viability (lead compounds) were highlighted, namely, (2) quercetin 3-galactoside (Fig. 10d), (4) tricetin 3’-xyloside (Fig. 10e), (6) kaempferol 4’-glucoside (Fig. 10c), (12) (-)-catechin gallate (Fig. 10a), and (14) epigallocatechin gallate (Fig. 10b). The controls amphotericin B and fluconazole exhibited affinity energies of -10.3 kcal/mol and − 8.1 kcal/mol, respectively. Our prioritization logic was designed to move beyond simple binding affinity by incorporating structural maturity and safety profiles. For instance, Amentoflavone demonstrated the highest binding affinity against CnFTase (-11.6 kcal/mol), yet it was excluded because its MCE-18 score (36.00) indicates a lack of structural complexity required for modern drug candidates. Furthermore, it was identified as a CYP2C9 substrate with a high risk of forming reactive quinone metabolites and a high probability (> 0.7) of endocrine disruption. Similarly, highly complex molecules like Citbismine C (MCE-18 = 148.00) were excluded due to predicted metabolic instability and poor synthetic accessibility. Only compounds that balanced high affinity with safe pharmacokinetic profiles and optimal structural complexity (MCE-18 between 63 and 100) were progressed as lead candidates.

**Fig. 11:**
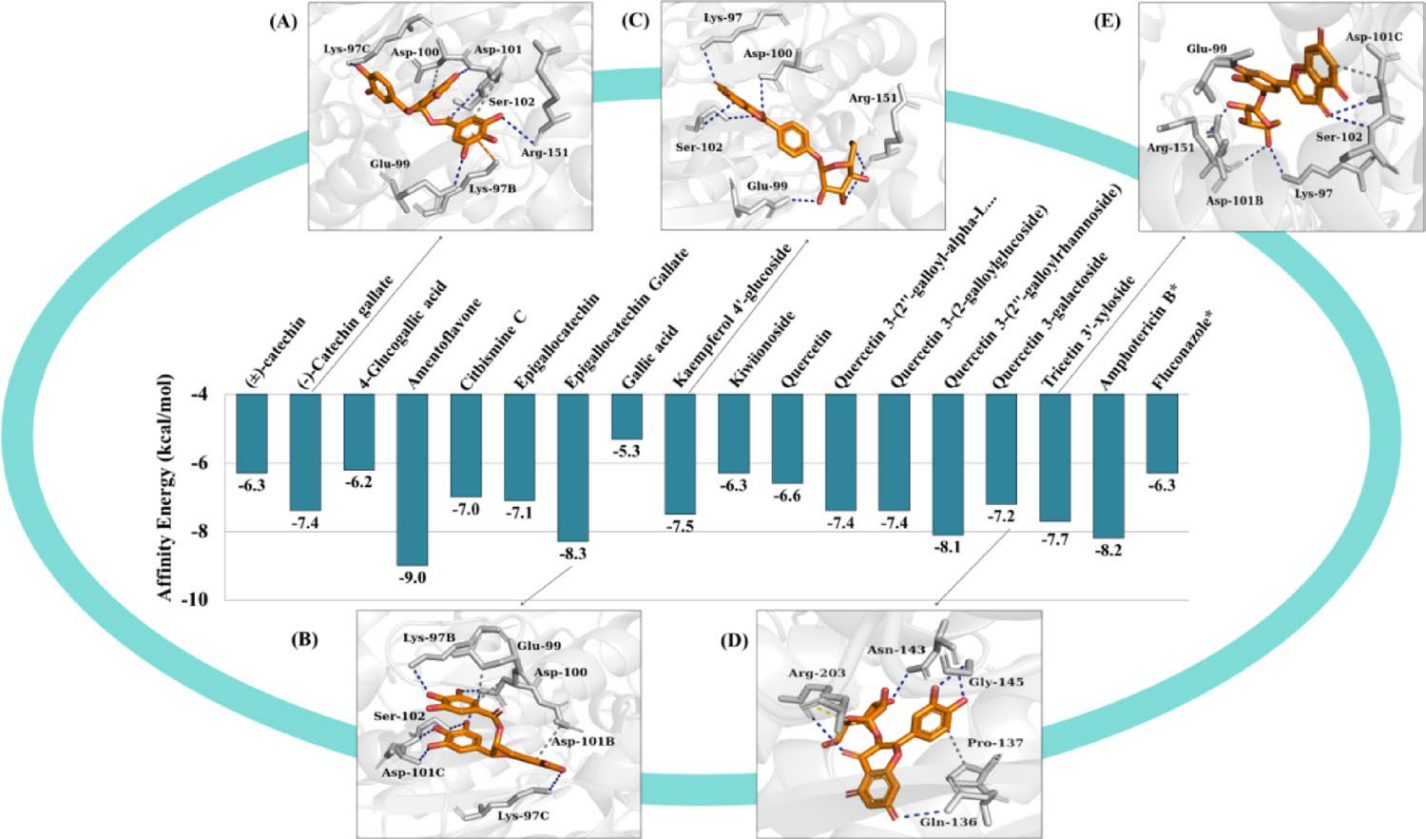
Affinity energy (kcal/mol) of the compounds against β-CA

**Fig. 12:**
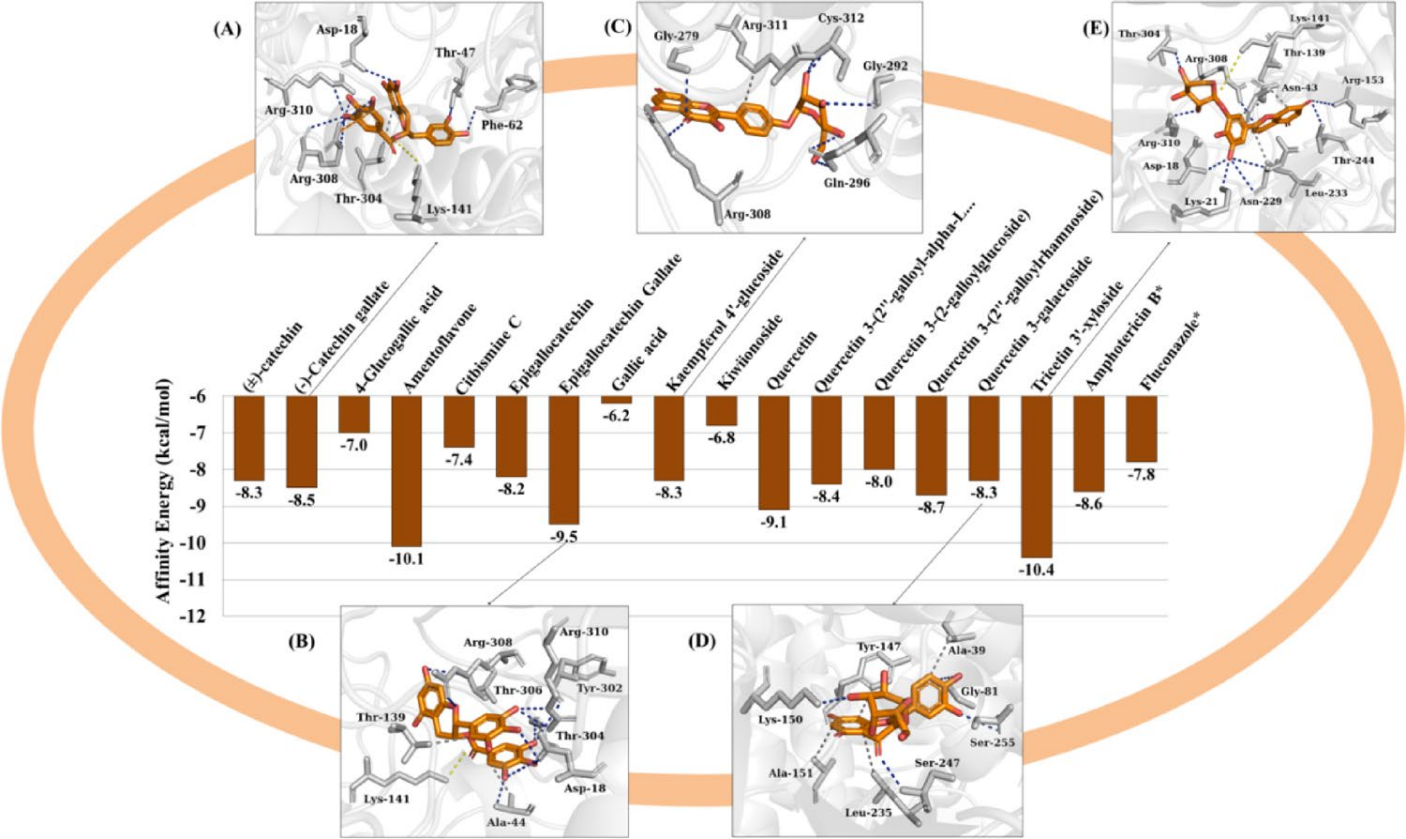
Affinity energy (kcal/mol) of the compounds against AdSS

With respect to β-CA, compound (2) exhibited an affinity energy of -7.2 kcal/mol (Fig. 11d), (4) demonstrated − 7.7 kcal/mol (Fig. 11e), (6) showed − 7.5 kcal/mol (Fig. 11c), and (12) presented − 7.4 kcal/mol (Fig. 11a) and (14) showed a value of -8.3 kcal/mol (Fig. 11b), while the controls amphotericin B and fluconazole showed affinity energies equal to -8.2 kcal/mol and − 6.3 kcal/mol, respectively.

In relation to AdSS, we observed that the lead compounds had favorable affinity energies, where compound (2) had an affinity energy of -8.3 kcal/mol (Fig. 12D), (4) -10.4 kcal/mol (Fig. 12E), (6) -8.3 kcal/mol (Fig. 12C), (12) -8.5 kcal/mol (Fig. 12A) and (14) showed a value of -9.5 kcal/mol (Fig. 12B). The amphotericin B and fluconazole controls showed affinity energies of -8.6 kcal/mol and − 7.8 kcal/mol, respectively.

### Interactions between the phytochemicals and the targeted enzymes

**Fig. 13:**
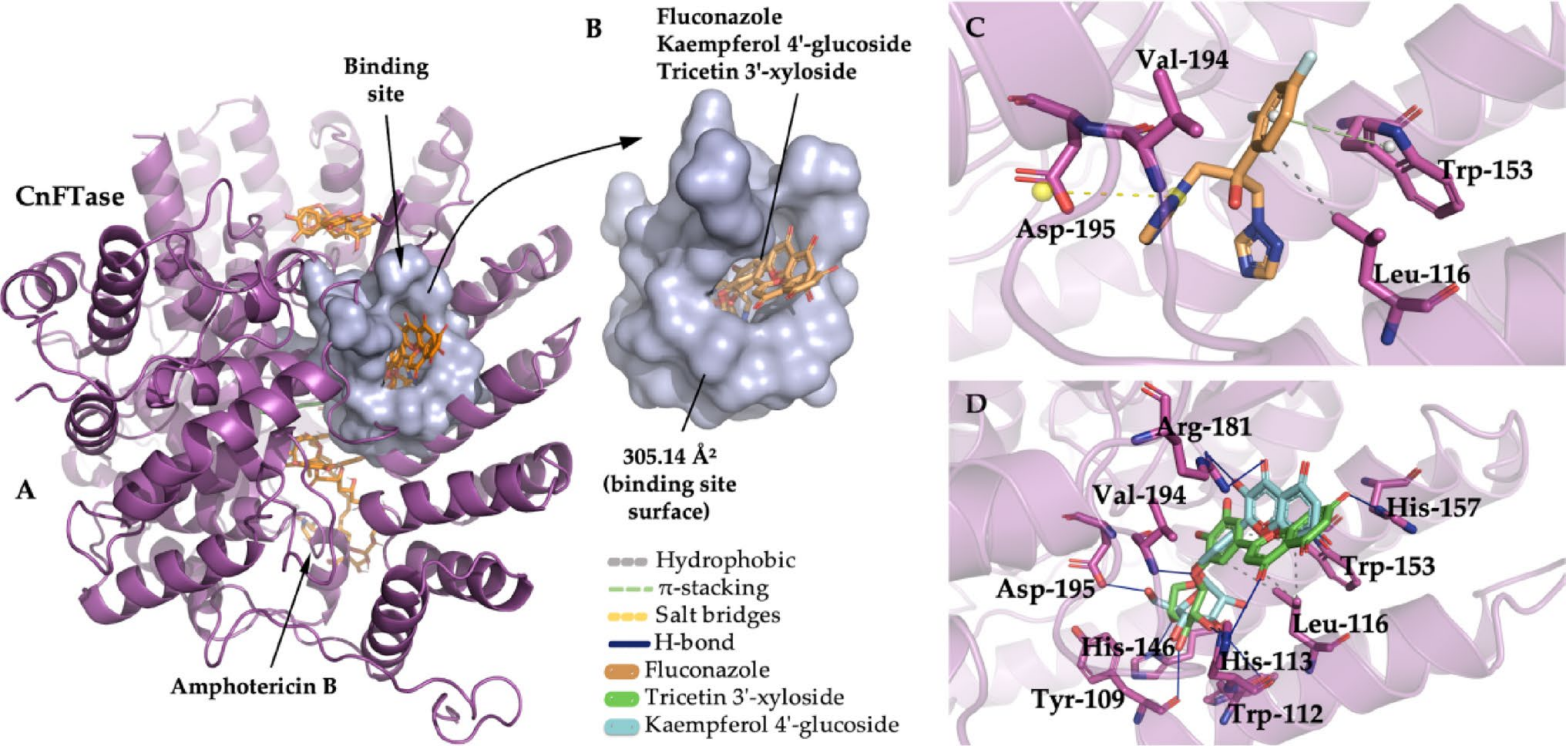
Three-dimensional image of the CnFTase protein (**A**), the binding cavity (**B**), binding site of fluconazole (**C**), binding sites of tricetin 3’-xyloside, kaempferol 4’-glucoside (**D**)

CnFTase is imperative for the virulence of *C. neoformans*, thus offering potential avenues for the development of specific FTase inhibitors to treat infectious disease species. Farnesylation is a prerequisite for the infectivity of the fungus (Wang et al. 2022). Molecular docking results showed that tricetin 3’-xyloside and kaempferol 4’-glucoside complexed in the same binding site as the control ligand fluconazole (Fig. 13a), where the *E*_A_ of less than − 6.0 kcal/mol in the formation of the ligand-protein complexes suggests that the phytochemicals show favorable specificity for the predicted cavity (Shityakov and Foerster 2014). The predicted protein cavity has a molecular surface area of 315.14 Å^2^, resulting from the side chain of amino acid residues, which include Tyr-109, Trp-112, His-113, Tyr-145, His-146, Tyr-150, Trp-153, Arg-181, Glu-193, Phe-223, Cys-236, Ala-237, Ser-238, Phe-239, Pro-240, Met-262, Tyr-269 (Fig. 13b), where the predominance of apolar and aromatic side chain residues constitute a hydrophobic cavity (Persch et al. 2015).

An analysis of the ligand-protein interactions indicates that the phytochemicals exhibited a series of interactions analogous to those observed in the fluconazole control, particularly with residues Leu-116 A, Trp-153 A, and Asp-195B (kaempferol 4’-glucoside). Leu-116 A, Val-194B (tricetin 3’-xyloside), indicating a similar action to the drug used (Fig. 13c and d), these being the lead compounds with the greatest specificity for the CnFTase active site.

**Fig. 14:**
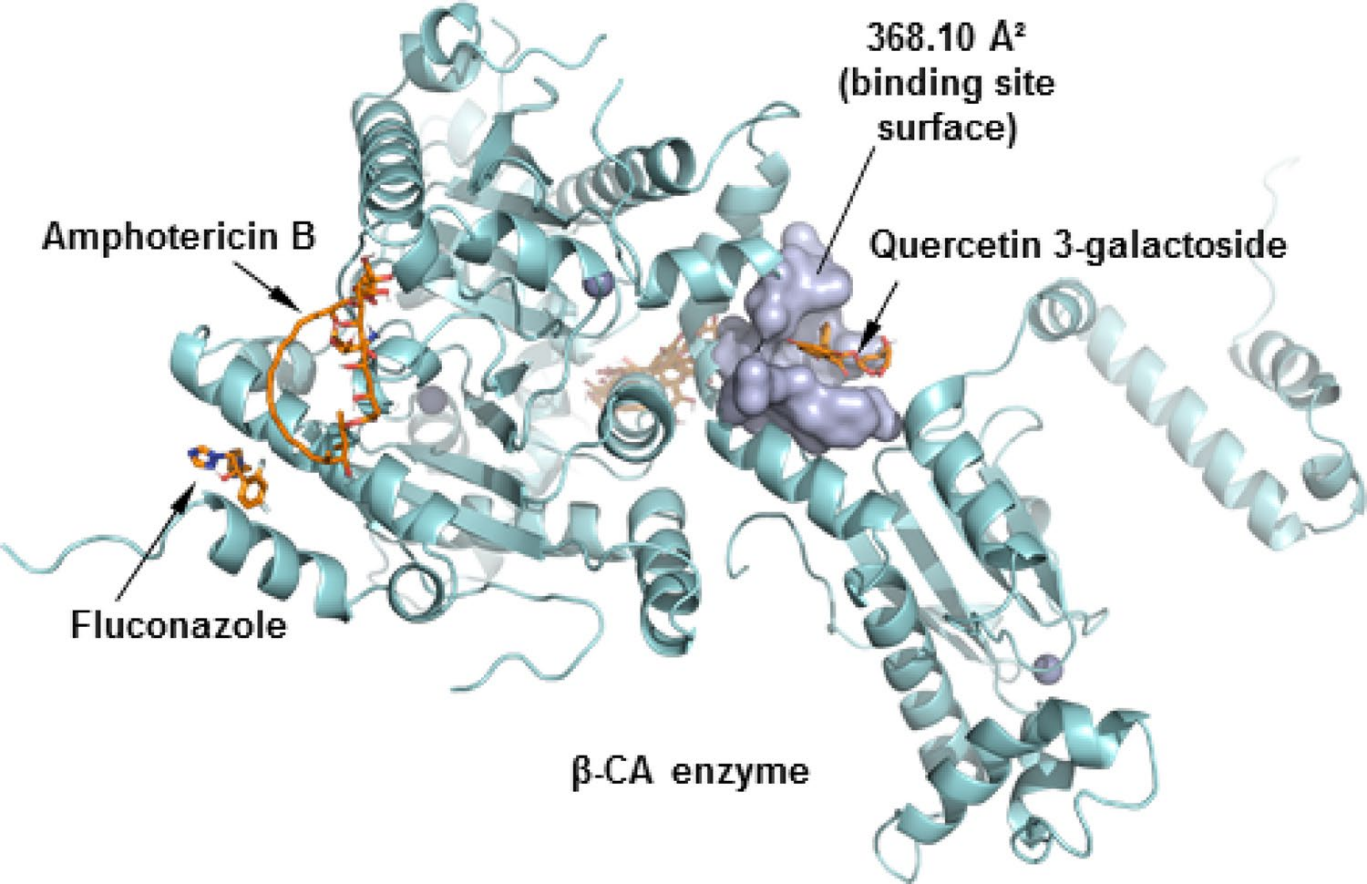
Three-dimensional image and binding cavity of the β-CA

The presence of β-CA has been observed in various fungal species. The CAN2 gene, which encodes β-carbonic anhydrase in *C. neoformans*, has been identified as being essential for growth in ambient conditions. However, this gene is dispensable for proliferation and virulence in vivo at high levels of CO_2_ in the host (Zhao et al. 2023). In the pathogenic fungus *C. neoformans*, the CO_2_-sensing system is essential for survival in the natural environment (approximately 0.03% CO_2_). This system mediates the switch to virulent growth in the human host (approximately 5% CO_2_) (Rathore et al. 2022). The molecular docking results demonstrated that only quercetin 3-galactoside remained in the predicted cavity, distant from the binding sites of the fluconazole and amphotericin B controls. This suggests that the isolated administration of the compound might not be sufficient to reverse the process of fungal proliferation under environmental conditions (Fig. 14).

**Fig. 15:**
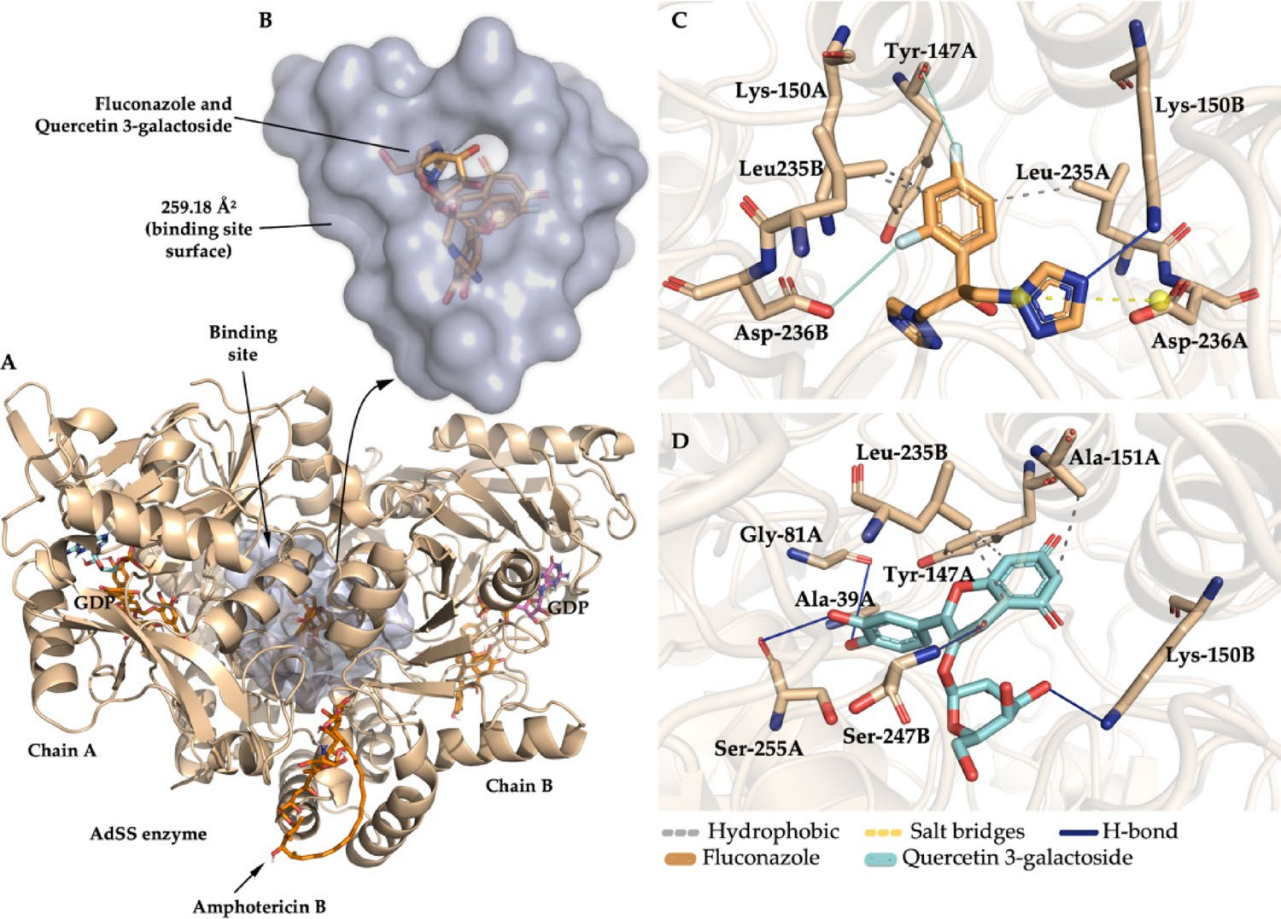
**a** Three-dimensional image of the AdSS protein, **b** the binding cavity, **c** binding site of fluconazole, **d** binding site of quercetin 3-galactoside

AdSS is a crucial enzyme in the ATP (adenosine triphosphate) biosynthetic pathway, catalyzing the magnesium-dependent formation of GTP (Blundell et al. 2016). The molecular docking results suggest that, among the lead compounds, quercetin 3-galactoside complexed to the AdSS binding site where the fluconazole control is located, indicating that the compound may present inhibitory activity similar to the drug (Fig. 15A), reinforced by the *E*_A_ lower than − 6.0 kcal/mol (Shityakov and Foerster 2014). The binding site was estimated to form a cavity with a molecular surface area of 259.18 Å^2^, located between chains A and B (Fig. 15B). This includes the amino acid residues between the interactions with the phytochemical and the control, i.e., Tyr-147 A, Lys-150B, Leu-235B.

In the analysis of ligand-protein interactions, it was observed that fluconazole has a fluoro-substituted aromatic moiety that contributes to the formation of a hydrophobic interaction with the apolar side chain of Leu-235B (Fig. 15C), as does quercetin 3-galactoside through its aromatic chromone ring (Fig. 15D), suggesting that substituted aromatic moieties are pharmacophores with greater specificity for forming hydrophobic interactions with the AdSS inhibition site (Blundell et al. 2016; Fokoue et al. 2020). In addition, quercetin 3-galactoside formed an H-bond interaction with the hydrogen donor part of the Lys-150B side chain, with a strong contribution from one of the OH groups of the galactoside substructure (Fig. 15D), just as the heteroaromatic substructure of fluconazole serves as an H-bond acceptor site for the same part of Lys-150B (Fig. 15C). The other lead compounds are not inserted into the interaction cavity and show less specificity for AdSS.

### Molecular dynamics simulations

**Fig. 16:**
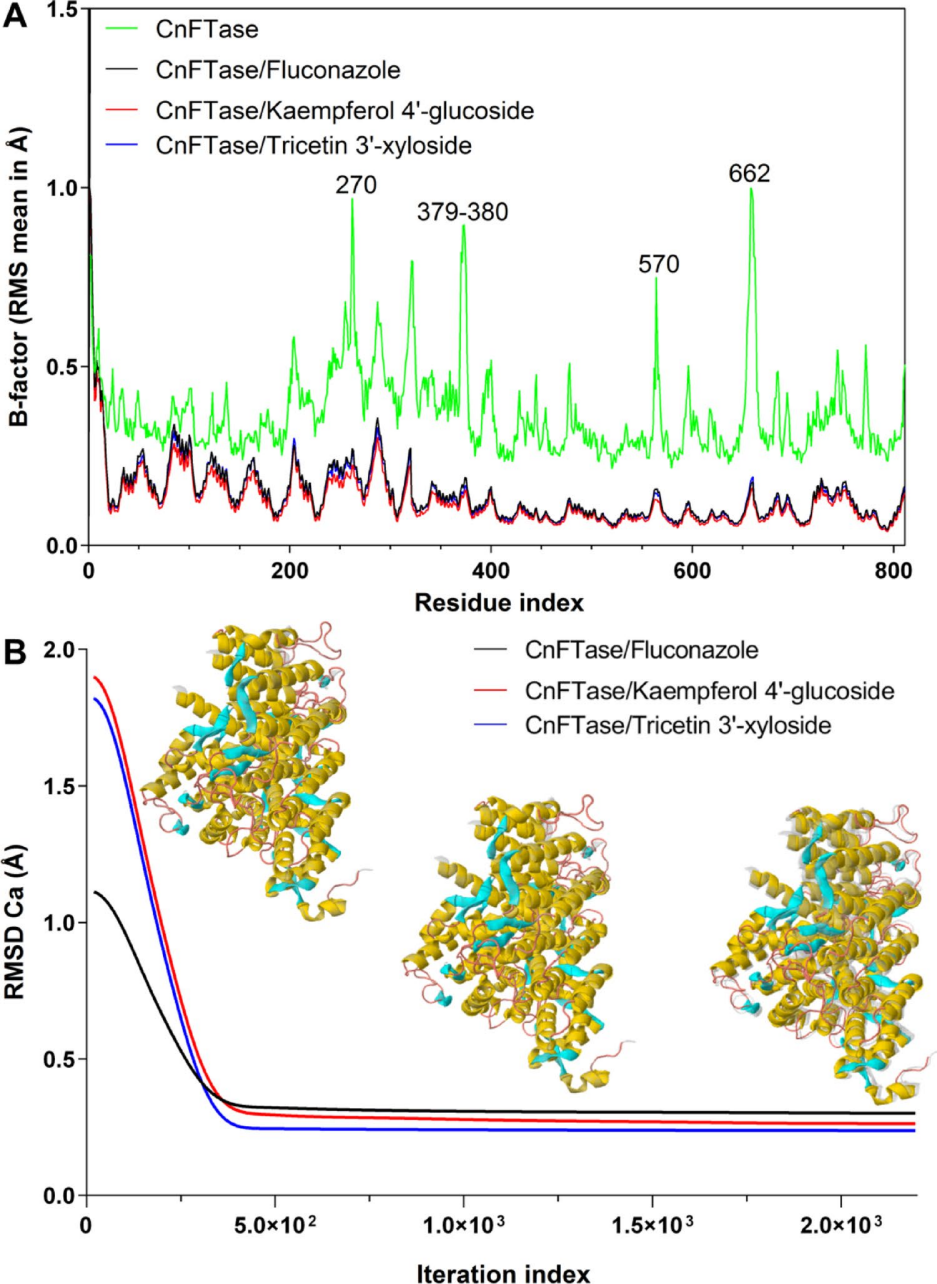
**A** B factors (RMS mean in Å) derived from normal mode analysis (NMA) showing flexible regions of the CnFTase (green line), highlighting the residues 270, 379–380, 570, and 662, indicating that both ligands (Fluconazole, Kaempferol 4’-glucoside, and Tricetin 3’-xyloside) similarly stabilize the receptor. **B** Convergence of the morphing trajectory for the complexes, monitored by the RMSD of Cα atoms throughout the iterations, the CnFTase-Fluconazole complex converged to a final RMSD of 0.301 Å, CnFTase-Kaempferol 4’-glucoside converged to a final RMSD of 0.256 Å, while the CnFTase-Tricetin 3’-xyloside converged to 0.236 Å, both with ∼2.200 × 10^3^ iterations

Flexibility is a critical factor for the interaction protein-ligand (Ghosh et al. 2021). iMODS analyses the molecular motion in addition to the structural flexibility via NMA (Normal mode analysis), which is incorporated with the coordinates of the docked complex (López-Blanco et al. 2014). The B-factor is associated with the protein’s mobility (flexibility) (Kovacs et al. 2004). The B-factor analysis (Fig. 16A) of CnFTase (green line) generated an average RMS, observed to have peaks with a flexibility index of approximately 1.0 Å for residues 270, 379–380, 570, and 662. Whereas CnFTase-Fluconazole (black line), CnFTase-Kaempferol 4’-glucoside (red line), and CnFTase-Tricetin 3’-xyloside (blue line) produced an significant low and an average RMS in the B-factor, indicating decreased flexibility of the residues in CnFTase after the formation of protein-ligand complexes with RMS mean values that are lower than 0.5 Å (Fig. 16A).

In this NMA trajectory, it is evident that, despite their stability in relation to the CnFTase (green line), the Kaempferol 4’-glucoside (red line) exhibited enhanced stability in terms of low conformational flexibility when compared to the Fluconazole (black line). This observation aligns with the energy state of affinity observed in molecular docking simulations, where Kaempferol 4’-glucoside it interacts at the same site as Fluconazole with an affinity energy of -9.6 kcal/mol, while fluconazole showed an affinity energy of -8.1 kcal/mol (Supplementary Table S1).

Upon analysis of the iteration trajectory, it was ascertained that the NMA mode indicated a smaller conformational torsion of the Cα of the CnFTase structure at the inception and conclusion of the MD simulations (Fig. 16B). In the trajectory of the CnFTase-Fluconazole complex (black line), it is possible to observe an initial RMSD of approximately 1.063 Å, with convergence iteration of approximately 2.200 × 10^3^ (calculation step), resulting in an last RMSD of 0.301 Å (Fig. 16B). The calculated value up to 2.0 Å expresses a plausible physiological movement, without exaggeration or structural collapse (López-Blanco et al. 2014; da Rocha et al. 2024b). For the CnFTase-Kaempferol 4’-glucoside complex (red line), an initial RMSD of approximately 1.777 Å is observed, where the structure stabilizes with an RMSD of 0.256 Å and convergence iteration in the order of 2.200 × 10^3^ (Fig. 16B). For the CnFTase-Tricetin 3’-xyloside complex (blue line), an initial RMSD of approximately 1.696 Å is observed, where the structure stabilizes with an RMSD of 0.236 Å and convergence iteration in the order of 2.200 × 10^3^ (Fig. 16B). The results of the NMA-based MD simulations suggest that collective movements for both complexes are stable.

**Fig. 17:**
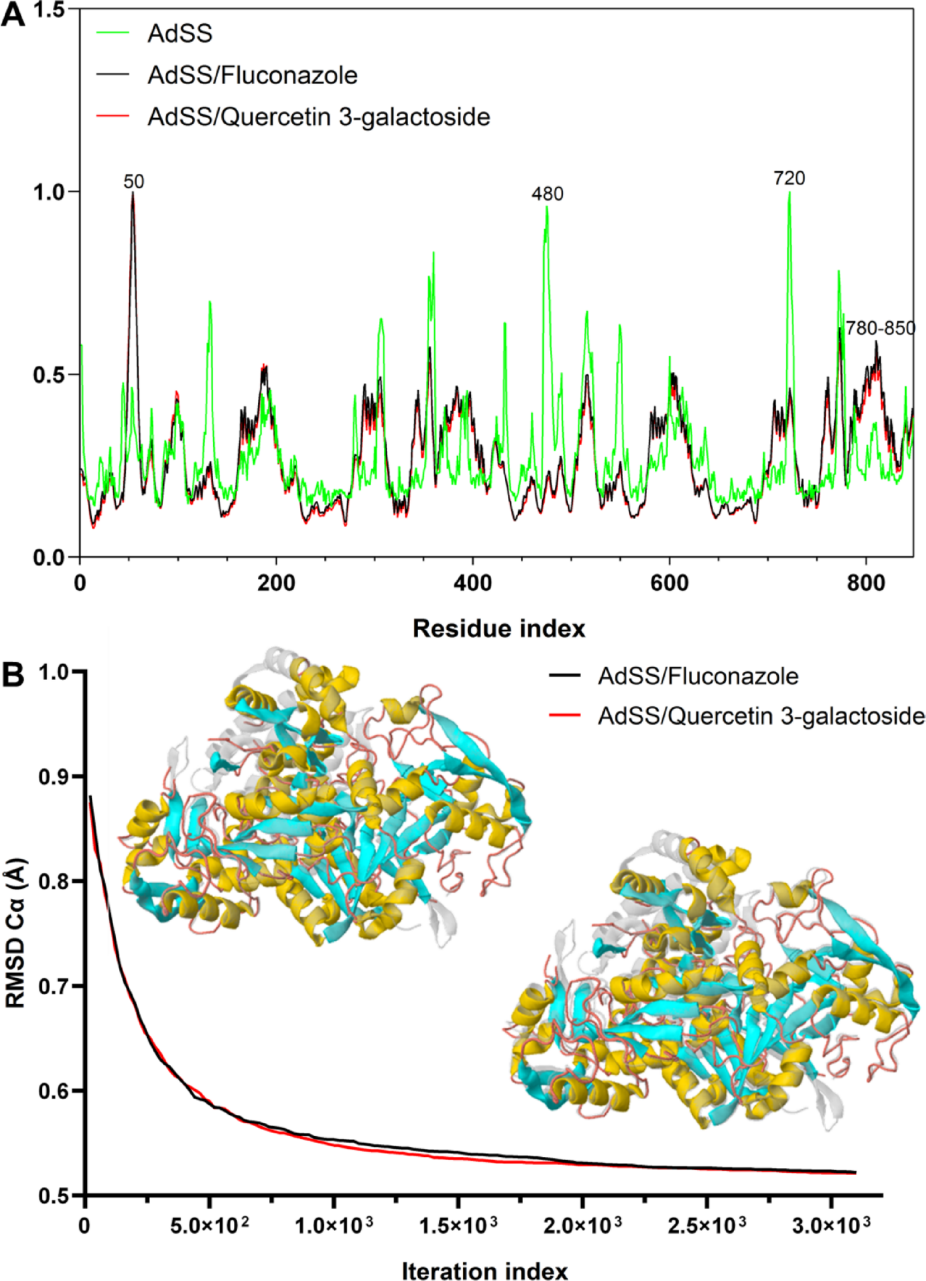
**A** B factors (RMS mean in Å) derived from normal mode analysis (NMA) showing flexible regions of the AdSS, highlighting the residues 50, 480, 720, and 780–850, indicating that both ligands (Fluconazole and Quercetin 3-galactoside) similarly stabilize the receptor. **B** Convergence of the morphing trajectory for the complexes, monitored by the RMSD of Cα atoms throughout the iterations, the AdSS-Fluconazole complex converged to a final RMSD of 0.519 Å, while the AdSS-Quercetin 3-galactoside converged to a final RMSD of 0.521 Å, both with ∼3.100 × 10^3^ iterations

The B-factor analysis (Fig. 17A) of AdSS (green line) generated an average RMS, observed to have peaks with a flexibility index of approximately 1.0 Å for residues 480 and 720. Whereas AdSS-Fluconazole (black line) and AdSS-Quercetin 3-galactoside (red line) produced an average RMS lower in the B-factor, indicating decreased flexibility of the residues in AdSS after the formation of protein-ligand complexes, except residue 50 and residue sequence 780–850 (Fig. 17A).

Upon analysis of the iteration trajectory, Quercetin 3-galactoside exhibits similar action to Fluconazole against AdSS with an initial RMSD in the order of 0.920 Å. The final RMSD of AdSS-Fluconazole e AdSS-Quercetin 3-galactoside complexes decreased to a value to 0.519 Å and 0.521Å respectively, with convergence iteration of approximately 3.100 × 10^3^ (calculation step), indicating a smaller conformational torsion of the Cα of the receptor structure (Fig. 17B). For these simulations, RMSD values lower than 3.0 Å suggest that the conformational changes are not drastic and, therefore, are within the ideality limit that provides the stable formation of the complexes (López-Blanco et al. 2014).

The primary goal of employing NMA in this study was to conduct an exploratory evaluation of the conformational stability and the collective motions of the receptor-ligand complexes. Our findings regarding the stability of the complexes are specifically limited to these large-scale collective behaviors. The convergence of RMSD values (0.236–0.301 Å for CnFTase and 0.519–0.521 Å for AdSS) indicates that the protein-ligand complexes exhibit stable collective movements within plausible physiological limits, without structural collapse. The B-factor analysis reflects the flexibility of the protein backbone (Cα), showing that the binding of lead phytochemicals such as kaempferol 4’-glucoside and quercetin 3-galactoside effectively stabilizes the target structures.

NMA provides a robust exploratory framework for assessing the conformational stability and collective motions of the CnFTase and AdSS complexes. The simulations are based in the ENM, which simplifies the protein structure into a system of masses and springs to capture large-scale mechanical movements. This approach successfully demonstrated that the binding of lead compounds-such as kaempferol 4’-glucoside and quercetin 3-galactoside-results in stable complexes with RMSD values well below the physiological threshold (0.2–0.5 Å) and significantly reduced B-factors, it does not substitute for full atomistic molecular dynamics simulations that account for solvent effects and individual atomic interactions. Consequently, these results serve as a computational validation of the docking stability, and the predicted antifungal potential of these cashew leaf phytochemicals.

## Conclusions

The integrative approach combining in silico pharmacokinetic and pharmacodynamic prediction techniques demonstrated that the compounds may be promising against the pathogenic fungus, with emphasis on the five lead compounds selected in the pharmacokinetic study: (2) quercetin 3-galactoside, (4) tricetin 3’-xyloside, (6) kaempferol 4’-glucoside, (12) (-)-catechin gallate and (14) epigallocatechin gallate. The molecular docking data demonstrated that the compounds exhibited significant affinity energies against CnFTase and AdSS, suggesting their capacity to act as inhibitors, in addition to interacting with amino acid residues present in the binding site of fluconazole (control) and the predicted cavity. Molecular dynamics simulations indicated a smaller conformational torsion of the Cα of the CnFTase and AdSS structures, suggesting that collective movements for both protein-ligand complexes are stable. Further in vitro and in vivo research will be needed to validate its antifungal effectiveness precisely.

## Supplementary Information

Below is the link to the electronic supplementary material.

Supplementary Material 1

## Data Availability

All the data generated or analyzed during this study are included in this published article.

## Abbreviations

Acyc: Acyclic
ADME: Absorption, distribution, metabolism and excretion
AdSS: Adenylosuccinate synthetase
AhR: Aryl hydrocarbon receptor
AI: Artificial intelligence
AnR: Androgen receptor
AnR-LBD: Androgen receptor ligand binding domain
AR: Aromatic ring
Caco-2: Adenocarcinoma colorectal cell
*Cl*_Hepa_: Clearance in hepatocytes
*Cl*_int_: Intrinsic hepatic clearance
*Cl*_Micro_: Clearance in liver microsomes
CnFTase: Farnesyltransferase
CNS: Central Nervous System
Cyc: Cyclic
CYP450: Cytochrome P450
DP: *Daphnia magna*
EA: Affinity Energy
EC: Eye corrosion
EI: Eye irritation
ER: Aromatase, estrogen receptor alpha
ER-LBD: Estrogen receptor ligand binding domain
FM: Fathead minnow
FPPL: Fungal priority pathogens list
Fsp3: Fraction sp3
P-gp: P-glycoprotein
H-HT: Hepatotoxicity
IGC_50_: Growth inhibition concentration
IP: Intraperitoneal
IV: Intravenous
LC_50_: Lethal concentration of 50% of a specie
LD_50_: Lethal dose of 50% of a specie
LGA: Lamarckian genetic algorithm
logP: Intrinsic lipophilicity
MCE18: Medicinal chemistry evolution, 2018
MDCK: Madin-Darby Canine Kidney cells
MLP: Molecular lipophilicity potential
MMFF94: Merck molecular force field
MW: Molecular weight
NAR: Non-aromatic ring
PAMPA: Parallel artificial membrane permeability assay
*P*_app_: Apparent permeability
PDB: Protein data bank
PPAR-Gamma: Peroxisome proliferator-activated receptor gamma
RMSD: Root mean square deviation
ROA: Rat oral acute toxicity
SC: Subcutaneous
SMILES: Bioconcentration factor
TP: *Tetrahymena pyriformis*
TPSA: Topological Polar Surface Area
*V*_dss_: Volume of distribution
WHO: World Health Organization
β-CA: Beta-carbonic anhydrase
NMA: Normal mode analysis
ENM: Elastic network model
