# Supplementary material for: In silico analysis of Anacardium occidentale phytochemicals: pharmacokinetics, molecular docking, and dynamics of Cryptococcus neoformans enzymes
Source: In Silico Pharmacol. 2026 Feb 26;14(1):84. doi: 10.1007/s40203-026-00590-y (PMC12936328; doi:10.1007/s40203-026-00590-y)
Supplement: Supplementary file 1 — Supplementary Material 1 [file 40203_2026_590_MOESM1_ESM.docx]

**In Silico Investigation of *Anacardium occidentale* Leaves Phytochemicals: MCE-18 Based Pharmacokinetics Analysis, Molecular Docking and Dynamics Simulations Targeting *Cryptococcus neoformans* CnFTase, β-CA and AdSS enzymes**

**Supplementary Data**

**Table S1. RMSD and types of interactions formed between ligands and the CnFTase.**

| **Ligand** | **RMSD (Å)** | **Interaction Type** | **Residue (Distance in Å)** |
| --- | --- | --- | --- |
| **(±)-catechin** | 1.729 | Hydrophobic | Tyr-109A (3.63), Glu-193B (3.99) |
|  |  | H-Bond | Arg-181B (1.99), Arg-181B (2.30), Asp-195B (2.33) |
| **(-)-Catechin gallate** | 0.085 | Hydrophobic | Ala-226B (3.73), Gln-228B (3.61), Leu-290B (3.74) |
|  |  | H-Bond | Lys-222B (2.85), Thr-229B (3.11), Arg-259B (1.90), Arg-259B (2.69), Ser-261B (2.36), Ser-261B (3.27) |
| **4-Glucogallic acid** | 1.190 | Hydrophobic | Val-194B (3.89), Ala-237B (3.77) |
|  |  | H-Bond | Trp-112A (2.49), His-113A (2.89), His-146A (2.06), Val-194B (2.16), Asp-195B (3.19) |
|  |  | Salt Bridges | Arg-181B (4.39) |
| **Amentoflavone** | 1.397 | Hydrophobic | Leu-84B (3.55), Trp-94B (3.84), Tyr-109A (3.92), Tyr-409B (3.83), Tyr-409B (3.63), Tyr-409B (3.99) |
|  |  | H-Bond | Ser-87B (3.63), Gln-110A (3.41), Arg-197B (2.41), Asp-407B (2.55), Tyr-409B (3.38), His-410B (3.26) |
| **Citbismine C** | 1.463 | H-Bond | Arg-235A (2.68), Lys-239A (3.01), Arg-329A (2.67), Arg-329A (3.16) |
|  |  | π-Cation | Arg-235A (5.63) |
| **Epigallocatechin** | 1.631 | Hydrophobic | Leu-116A (3.63), Ala-149A (3.79), Ala-237B (3.97) |
|  |  | H-Bond | Tyr-109A (2.84), His-146A (2.13), Trp-153A (3.58), Arg-181B (2.32), Ser-238B (3.23) |
| **Epigallocatechin Gallate** | 0.258 | Hydrophobic | Tyr-109A (3.75), Tyr-109A (3.47), Leu-141B (3.46) |
|  |  | H-Bond | Tyr-109A (2.91), Gln-110A (2.86), His-146A (2.54) |
|  |  | π-Cation | Arg-197B (5.69) |
|  |  | Salt Bridges | Arg-197B (5.16) |
| **Gallic acid** | 0.068 | Hydrophobic | Glu-193B (3.97) |
|  |  | H-Bond | His-113A (3.04), His-146A (1.97), Val-194B (3.13), Val-194B (2.63), Asp-195B (2.14), Tyr-269B (2.41), Tyr-269B (3.09) |
| **Kaempferol 4'-glucoside** | 0.933 | Hydrophobic | Leu-116A (3.87), Trp-153A (3.79) |
|  |  | H-Bond | Trp-112A (3.67), His-113A (2.41), Arg-181B (2.96), Arg-181B (2.86), Asp-195B (2.12) |
| **Kiwiionoside** | 1.490 | H-Bond | Trp-194A (3.32), Ala-226B (3.32), Thr-229B (2.44), Ser-261B (2.67), Asp-293B (2.82) |
|  |  | Salt Bridges | His-240A (5.41) |
| **Quercetin** | 1.786 | Hydrophobic | Tyr-109A (3.70), Ala-149A (3.59), Trp-153A (3.86), Val-194B (3.92), Ala-237B (3.99) |
|  |  | H-Bond | His-113A (1.87), His-146A (2.15), Arg-181B (2.23), Arg-181B (2.71), Ser-238B (3.19), Tyr-269B (3.37) |
| **Quercetin 3-(2''-galloyl-alpha-L-arabinopyranoside)** | 1.929 | Hydrophobic | Trp-94B (3.79), Tyr-109A (3.93), Tyr-409B (3.75), Tyr-409B (3.95) |
|  |  | H-Bond | Ala-72A (2.92), Ser-87B (3.03), Tyr-109A (2.68), Gln-110A (2.54), Arg-197B (2.07), Arg-197B (3.03) |
| **Quercetin 3-(2-galloylglucoside)** | 1.794 | Hydrophobic | Ala-226B (3.71), Gln-228B (3.93), Arg-259B (3.96), Val-260B (3.92) |
|  |  | H-Bond | Trp-194A (2.24), Val-198A (2.67), Ser-225B (2.02), His-240A (3.33), Arg-259B (3.62), Arg-259B (2.11), Ser-261B (2.51) |
| **Quercetin 3-(2''-galloylrhamnoside)** | 1.976 | Hydrophobic | Ala-72A (3.73), Trp-329B (3.79), Tyr-409B (3.46), Tyr-409B (3.71) |
|  |  | H-Bond | Ala-72A (3.11), Ser-87B (2.42), Gln-110A (2.12), Tyr-326B (3.14) |
|  |  | π-Stacking | Trp-90B (5.35), Trp-90B (5.00) |
| **Quercetin 3-galactoside** | 1.843 | Hydrophobic | Gln-228B (3.70) |
|  |  | H-Bond | Trp-194A (2.00), Ser-225B (1.89), Thr-229B (3.07), Gly-236A (2.87), His-240A (2.82), Asp-293B (2.97) |
| **Tricetin 3'-xyloside** | 1.405 | Hydrophobic | Leu-116A (3.87), Leu-116A (3.70) |
|  |  | H-Bond | Tyr-109A (2.85), His-113A (3.62), His-113A (2.34), His-146A (1.90), His-157A (2.58), Val-194B (3.07) |
| **Amphotericin B*** | 1.072 | Hydrophobic | Ala-72A (3.69), Trp-94B (3.26), Tyr-109A (3.78), Arg-405B (3.99), Tyr-409B (3.40) |
|  |  | H-Bond | Ser-83B (3.22), Ser-87B (2.30), Lys-107A (2.80), Lys-107A (1.99), Arg-405B (2.69), Asp-407B (1.78), |
|  |  | Salt Bridges | Lys-80B (4.81) |
| **Fluconazole*** | 1.119 | Hydrophobic | Leu-116A (3.89) |
|  |  | H-Bond | Val-194B (3.19) |
|  |  | π-Stacking | Trp-153A (4.57) |
|  |  | Salt Bridges | Asp-195B (5.36) |

***Controls**

**Table S2. RMSD and types of interactions formed between ligands and the β-CA.**

| **Ligand** | **RMSD (Å)** | **Interaction Type** | **Residue (Distance in Å)** |
| --- | --- | --- | --- |
| **(±)-catechin** | 1.418 | H-Bond | Glu-99B (3.31), Glu-99B (3.41), Asp-100C (2.04), Asp-101B (2.28), Asp-101C (2.94), Ser-102C (2.26), Ser-102C (3.01) |
| **(-)-Catechin gallate** | 1.635 | Hydrophobic | Asp-100C (3.89), Asp-101C (3.71) |
|  |  | H-Bond | Lys-97C (2.30), Glu-99B (1.86), Asp-101C (1.91), Ser-102C (3.00), Arg-151B (2.74) |
|  |  | π-Cation | Lys-97B (3.97) |
| **4-Glucogallic acid** | 1.698 | Hydrophobic | Asp-101B (3.81) |
|  |  | H-Bond | Asp-100C (1.99), Asp-100C (2.31), Asp-101B (2.11), Asp-101B (2.55), Asp-101C (2.32), Ser-102C (2.58), Ser-102C (2.10), |
|  |  | Salt Bridges | Lys-97C (3.83) |
| **Amentoflavone** | 1.649 | Hydrophobic | Tyr-39C (3.70), Val-44B (3.76), Thr-77C (3.86) |
|  |  | H-Bond | Lys-43C (2.10), Glu-48B (2.29), Thr-77B (2.80) |
|  |  | π-Stacking | Trp-40B (5.04) |
| **Citbismine C** | 1.949 | Hydrophobic | Thr-77C (3.92) |
|  |  | H-Bond | Trp-40C (3.53), Lys-43C (3.23), Val-76C (2.30) |
|  |  | π-Stacking | Trp-40C (4.75) |
|  |  | π-Cation | Lys-43C (3.71) |
| **Epigallocatechin** | 0.144 | H-Bond | Lys-97C (1.94), Asp-100B (3.62), Asp-100B (3.38), Asp-101B (2.96), Asp-101B (2.32), Asp-101C (2.02), Asp-101C (2.93), Ser-102C (2.43), Ser-102C (2.14), Ser-102C (2.04), Ser-102C (2.55) |
| **Epigallocatechin Gallate** | 0.094 | Hydrophobic | Glu-99B (3.49), Asp-100B (3.99), Asp-101B (3.82) |
|  |  | H-Bond | Lys-97B (2.45), Lys-97C (2.96), Asp-100B (2.06), Asp-100B (2.06), Asp-101C (1.99), Ser-102C (2.31), Ser-102C (1.98) |
| **Gallic acid** | 0.184 | Hydrophobic | Gln-104B (3.66), Pro-148C (3.70), Pro-188B (3.69), Pro-188B (3.52) |
|  |  | H-Bond | Asn-108B (2.79), Thr-189B (2.57), Glu-204A (2.21) |
|  |  | Salt Bridges | His-229A (4.60) |
| **Kaempferol 4'-glucoside** | 1.599 | H-Bond | Lys-97B (2.10), Glu-99C (2.28), Asp-100B (2.48), Ser-102C (3.33), Ser-102C (3.25), Arg-151C (3.21), Arg-151C (2.95) |
| **Kiwiionoside** | 1.907 | Hydrophobic | Glu-99C (3.70) |
|  |  | H-Bond | Lys-97B (3.11), Lys-97C (2.29), Glu-99B (2.70), Asp-101B (2.43), Asp-101C (3.18), Ser-102C (3.40) |
| **Quercetin** | 1.861 | Hydrophobic | Glu-13C (3.57), Thr-216B (3.92) |
|  |  | H-Bond | Glu-13C (2.84), Asp-17C (3.40), Arg-42C (2.71), Thr-45C (2.11) |
| **Quercetin 3-(2''-galloyl-alpha-L-arabinopyranoside)** | 1.683 | Hydrophobic | Lys-97B (3.80), Asp-101B (3.63) |
|  |  | H-Bond | Lys-97B (2.26), Lys-97C (2.21), Glu-99B (2.40), Glu-99B (2.61), Asp-101C (2.58), Ser-102C (2.09), Ser-102C (2.92), Arg-151B (2.20), Arg-151B (2.41) |
| **Quercetin 3-(2-galloylglucoside)** | 1.807 | H-Bond | Trp-40C (3.00), Glu-48C (2.34), Val-76B (2.98), Ala-80B (3.73), Arg-81B (2.32), Arg-81B (2.19), Arg-81C (1.77), Lys-82C (2.29) |
|  |  | π-Stacking | Trp-40C (4.77) |
| **Quercetin 3-(2''-galloylrhamnoside)** | 0.191 | Hydrophobic | Phe-51C (3.66), Glu-54C (3.50) |
|  |  | H-Bond | Glu-32B (2.70), Glu-48C (3.07), Glu-54C (2.72), Ala-60C (2.05), Ala-60C (3.03), Arg-81C (3.34) |
| **Quercetin 3-galactoside** | 1.215 | Hydrophobic | Pro-137C (3.96) |
|  |  | H-Bond | Gln-136C (2.28), Asn-143C (2.03), Asn-143C (2.04), Gly-145C (2.26), Arg-203A (2.85), |
|  |  | Salt Bridges | Arg-203A (4.74) |
| **Tricetin 3'-xyloside** | 1.768 | Hydrophobic | Glu-99B (3.97), Asp-101C (3.97) |
|  |  | H-Bond | Lys-97C (2.07), Asp-101B (2.15), Asp-101C (2.88), Ser-102C (2.31), Arg-151C (2.19) |
| **Amphotericin B*** | 1.403 | Hydrophobic | Glu-35C (3.89), Tyr-39C (3.63) |
|  |  | H-Bond | Glu-32C (3.21), Glu-32C (3.38), Trp-40B (1.96), Val-76B (2.70), Thr-77C (2.42), |
|  |  | Salt Bridges | Arg-81B (5.08) |
| **Fluconazole*** | 1.025 | Hydrophobic | Arg-38C (3.60), Arg-42C (3.76) |
|  |  | π-Cation | Arg-42C (4.38) |

***Controls**

**Table S3. RMSD and types of interactions formed between ligands and the AdSS.**

| **Ligand** | **RMSD (Å)** | **Interaction Type** | **Residue (Distance in Å)** |
| --- | --- | --- | --- |
| **(±)-catechin** | 1.941 | Hydrophobic | Tyr-147B (3.77), Lys-150B (3.97), Leu-235B (3.58) |
|  |  | H-Bond | Tyr-147A (3.05), Tyr-147B (2.21), Asp-236B (3.67), Ser-247B (3.16), Gly-251A (2.45), Ser-255A (2.68) |
| **(-)-Catechin gallate** | 1.179 | Hydrophobic | Thr-304A (3.90) |
|  |  | H-Bond | Asp-18A (2.71), Thr-47A (2.38), Phe-62A (2.46), Arg-308A (3.03), Arg-308A (3.25), Arg-308A (3.07), Arg-310A (2.56), Arg-310A (2.31) |
|  |  | π-Cation | Arg-308A (4.50) |
|  |  | Salt Bridges | Lys-141A (3.94) |
| **4-Glucogallic acid** | 1.754 | H-Bond | Lys-150B (2.92), Asp-236B (2.23), Ser-247A (2.64) |
|  |  | π-Cation | Lys-150A (4.48) |
| **Amentoflavone** | 1.127 | Hydrophobic | Asn-43B (3.54), Ala-44B (3.80), Ala-44B (3.84), Thr-139B (3.60), Val-305B (3.32) |
|  |  | H-Bond | Asp-18B (2.30), Ala-44B (3.76), Ala-44B (2.09), Ser-88B (1.99), Asn-91B (1.80), Thr-304B (2.68), Val-305B (2.68), Thr-306B (3.48), Thr-306B (3.58), Arg-308B (2.57) |
| **Citbismine C** | 1.652 | Hydrophobic | Asp-208A (3.60) |
|  |  | H-Bond | Asp-111A (2.30), Ala-364B (2.38) |
|  |  | π-Cation | Arg-368B (5.78), Arg-368B (5.86) |
| **Epigallocatechin** | 1.422 | Hydrophobic | Thr-139B (3.56), Leu-233B (3.65), |
|  |  | H-Bond | Asp-18B (3.20), Asp-18B (3.22), Asp-18B (3.08), Lys-21B (3.20), Ala-44B (2.14), Gly-137B (1.76), Arg-308B (2.69), Arg-308B (2.47) |
| **Epigallocatechin Gallate** | 0.134 | Hydrophobic | Ala-44B (3.74), Thr-139B (3.46) |
|  |  | H-Bond | Asp-18B (3.42), Asp-18B (3.27), Asp-18B (2.22), Ala-44B (2.28), Tyr-302B (3.55), Thr-304B (2.13), Thr-306B (3.23), Arg-308B (2.71), Arg-310B (2.46), Arg-310B (2.40) |
|  |  | Salt Bridges | Lys-141B (3.55) |
| **Gallic acid** | 0.173 | Hydrophobic | Thr-138A (3.70) |
|  |  | H-Bond | Asn-43A (2.76), Thr-139A (2.91), Thr-139A (2.24), Arg-140A (3.25), Lys-141A (2.72), Ile-143A (3.03), Gly-144A (2.32), Arg-153B (3.37), Asn-229A (2.68), Thr-244A (3.56) |
|  |  | Salt Bridges | Arg-153B (5.32) |
| **Kaempferol 4'-glucoside** | 1.172 | Hydrophobic | Arg-311B (3.41) |
|  |  | H-Bond | Gly-279B (2.63), Gly-292B (3.09), Gln-296B (3.13), Gln-296B (3.16), Arg-308B (2.41), Arg-308B (2.31), Cys-312B (2.53), Cys-312B (3.04) |
| **Kiwiionoside** | 1.431 | Hydrophobic | His-159A (3.74), Phe-162A (3.98), Asp-365B (3.96) |
|  |  | H-Bond | Asp-208A (3.47), Asp-208A (1.84), Ala-364B (2.97), Asp-365B (2.48), Asp-365B (2.76), Arg-368B (2.65) |
|  |  | Salt Bridges | Arg-368B (5.05) |
| **Quercetin** | 0.620 | Hydrophobic | Asn-43B (3.34), Thr-139B (3.85), Leu-233B (3.53) |
|  |  | H-Bond | Trp-16B (2.34), Lys-21B (2.54), Asn-43B (2.34), Arg-153A (3.38), Asn-229B (2.12), Thr-244B (2.92), Val-278B (2.93) |
| **Quercetin 3-(2''-galloyl-alpha-L-arabinopyranoside)** | 1.902 | Hydrophobic | Ile-206A (3.74), Pro-363B (3.72) |
|  |  | H-Bond | Asp-111A (2.51), Ile-206A (2.43), Ala-364B (2.38), Asp-365B (2.50), Asp-365B (2.59) |
|  |  | π-Cation | Arg-368B (4.64) |
|  |  | Salt Bridges | Arg-203A (5.27), Arg-368B (5.03) |
| **Quercetin 3-(2-galloylglucoside)** | 1.950 | Hydrophobic | Ile-206A (3.77), Ile-206A (3.48), Asp-208A (3.73) |
|  |  | H-Bond | Arg-203A (2.79), Ile-206A (2.60), Glu-360B (2.54), Ala-364B (2.03), Asp-365B (3.15), Arg-368B (3.36) |
|  |  | π-Cation | Arg-368B (4.66) |
| **Quercetin 3-(2''-galloylrhamnoside)** | 1.461 | Hydrophobic | Ala-61A (3.63) |
|  |  | H-Bond | Asp-18A (2.69), Asn-43A (2.47), Thr-47A (2.90), Asn-63A (2.35), Thr-304A (2.54), Arg-308A (2.53), Arg-308A (2.76), Arg-310A (2.54), Arg-310A (3.26) |
|  |  | Salt Bridges | Lys-141A (5.39) |
| **Quercetin 3-galactoside** | 1.374 | Hydrophobic | Ala-39A (3.88), Tyr-147A (3.81), Ala-151A (3.84), Leu-235B(3.94) |
|  |  | H-Bond | Gly-81A (3.36), Lys-150B (2.65), Ser-247B (2.71), Ser-255A (2.60) |
| **Tricetin 3'-xyloside** | 1.668 | Hydrophobic | Asn-43A (3.76), Thr-139A (3.99), Leu-233A (3.37) |
|  |  | H-Bond | Asp-18A (2.41), Lys-21A (2.42), Arg-153B (2.97), Asn-229A (3.62), Asn-229A (3.26), Thr-244A (2.28), Thr-304A (2.19), Arg-308A (3.13), Arg-310A (2.45) |
|  |  | Salt Bridges | Lys-141A (4.70) |
| **Amphotericin B*** | 1.829 | Hydrophobic | Ile-354A (3.78), Arg-368A (3.63) |
|  |  | H-Bond | Asp-111B (2.85), Asp-111B (2.40), Glu-360A (3.17), Arg-368A (3.35), Arg-368A (2.71) |
|  |  | Salt Bridges | Arg-368A (4.91), Lys-371A (4.26) |
| **Fluconazole*** | 1.864 | Hydrophobic | Lys-150A (3.87), Leu-235A (3.79), Leu-235B (3.67) |
|  |  | H-Bond | Lys-150B (2.67) |
|  |  | Halogen Bond | Tyr-147A (3.15), Asp-236B (3.60) |
|  |  | Salt Bridges | Asp-236A (5.48) |

***Controls**
